# Endothelial lipid droplets suppress eNOS to link high fat consumption to blood pressure elevation

**DOI:** 10.1172/JCI173160

**Published:** 2023-12-15

**Authors:** Boa Kim, Wencao Zhao, Soon Y. Tang, Michael G. Levin, Ayon Ibrahim, Yifan Yang, Emilia Roberts, Ling Lai, Jian Li, Richard K. Assoian, Garret A. FitzGerald, Zoltan Arany

**Affiliations:** 1 Department of Pathology and Lab Medicine, McAllister Heart Institute, Nutrition Obesity Research Center, and Lineberger Cancer Center, University of North Carolina, Chapel Hill, North Carolina, USA.; 2 Department of Medicine, Cardiovascular Institute, and Institute of Diabetes Obesity and Metabolism, Perelman School of Medicine,; 3 Institute for Translational Medicine and Therapeutics, Perelman School of Medicine, and; 4 Department of Systems Pharmacology and Translational Therapeutics, University of Pennsylvania, Philadelphia, Pennsylvania, USA.

**Keywords:** Cardiology, Endothelial cells

## Abstract

Metabolic syndrome, today affecting more than 20% of the US population, is a group of 5 conditions that often coexist and that strongly predispose to cardiovascular disease. How these conditions are linked mechanistically remains unclear, especially two of these: obesity and elevated blood pressure. Here, we show that high fat consumption in mice leads to the accumulation of lipid droplets in endothelial cells throughout the organism and that lipid droplet accumulation in endothelium suppresses endothelial nitric oxide synthase (eNOS), reduces NO production, elevates blood pressure, and accelerates atherosclerosis. Mechanistically, the accumulation of lipid droplets destabilizes eNOS mRNA and activates an endothelial inflammatory signaling cascade that suppresses eNOS and NO production. Pharmacological prevention of lipid droplet formation reverses the suppression of NO production in cell culture and in vivo and blunts blood pressure elevation in response to a high-fat diet. These results highlight lipid droplets as a critical and unappreciated component of endothelial cell biology, explain how lipids increase blood pressure acutely, and provide a mechanistic account for the epidemiological link between obesity and elevated blood pressure.

## Graphical abstract

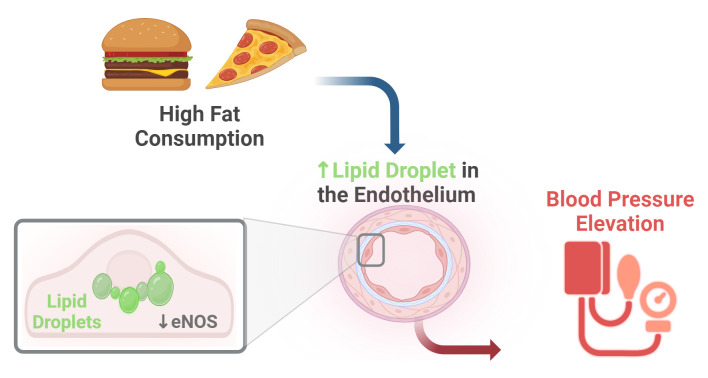

## Introduction

Numerous epidemiological studies demonstrate a strong connection between obesity and elevated blood pressure (BP) (1). Lipid loading in humans by i.v. infusion of lipids or by oral fat intake elevates BP acutely, demonstrating that lipid intake is causal to BP elevation (2–5). How lipids increase BP remains unclear.

BP is in large part regulated by the paracrine production of nitric oxide (NO) by endothelial NO synthase (eNOS) in endothelial cells (ECs). NO activates a complex cyclic GMP-dependent pathway in adjacent arteriolar smooth muscle cells, ultimately leading to cellular relaxation, reduced vascular resistance, and lower BP. The regulation of eNOS activity is complex and extensively studied, affected, for example, by insulin/Akt signaling, blood flow characteristics, availability of its substrate l-arginine, redox state, and reactive oxygen species, among others. How endothelial intracellular lipid homeostasis affects eNOS expression and activity has been less well studied, despite the vital role that the endothelium plays in lipid transport to underlying parenchyma. Infusion of free fatty acids (FFAs) in humans leads to reduced eNOS activity, NO production, and endothelium-dependent vasodilation, strongly implicating eNOS dysfunction in the connection between lipid homeostasis and BP (2–10). In ECs in culture, lipid ligands of PPARɑ can induce eNOS gene expression (11), while FFAs can suppress eNOS by various mechanisms (12–14). eNOS is also both myristoylated and palmitoylated, with important consequences on cellular localization and function (15, 16). The in vivo relevance of these cell culture findings is not certain, however; nor is whether or how they relate to BP regulation.

FFAs can be esterified and stored in triglyceride-rich (TG-rich) lipid droplets (LDs), canonically described in adipocytes, but also seen in other cell types. LDs have recently been recognized as more than inert storage sites for lipids, involved in addition in processes ranging from inflammation and cellular stress response to SARS-CoV-2 replication (17–19). Little is known about LDs in ECs, however. Recent work demonstrated that LDs can transiently accumulate in mouse aortic endothelium after oral gavage with olive oil (20). Incorporation and liberation of fatty acids (FAs) into and out of these LDs occurred via canonical enzymatic processes, including diacylglycerol O-acyltransferase (DGAT) and adipose triglyceride lipase (ATGL), respectively. Whether LDs also can be found in the vasoreactive microvasculature or in response to physiological lipid loading, such as high-fat feeding, was not addressed. The biologic consequence of LD accumulation in the endothelium, if any, also remains unknown.

Here, we demonstrate that ad libitum high-fat feeding is sufficient to promote accumulation of LDs in endothelium and that this occurs in both large and small vessels and is accompanied by elevated BP. We use endothelial deletion of ATGL in vivo to show that endothelial LDs are sufficient for BP elevation, and we show that suppression of LD formation in vivo lowers BP induced by high-fat feeding. Mechanistically, we show that LD accumulation suppresses eNOS, NO formation, and vascular reactivity and does so by destabilization of eNOS mRNA stability and by activation of a the NF-κB/ monocyte chemoattractant protein-1 (MCP1)/eNOS pathway. This work provides a mechanistic basis for the connection between obesity and elevated BP.

## Results

### High-fat diet promotes accumulation of LDs in endothelium and elevates BP in mice.

**Figure 1:**
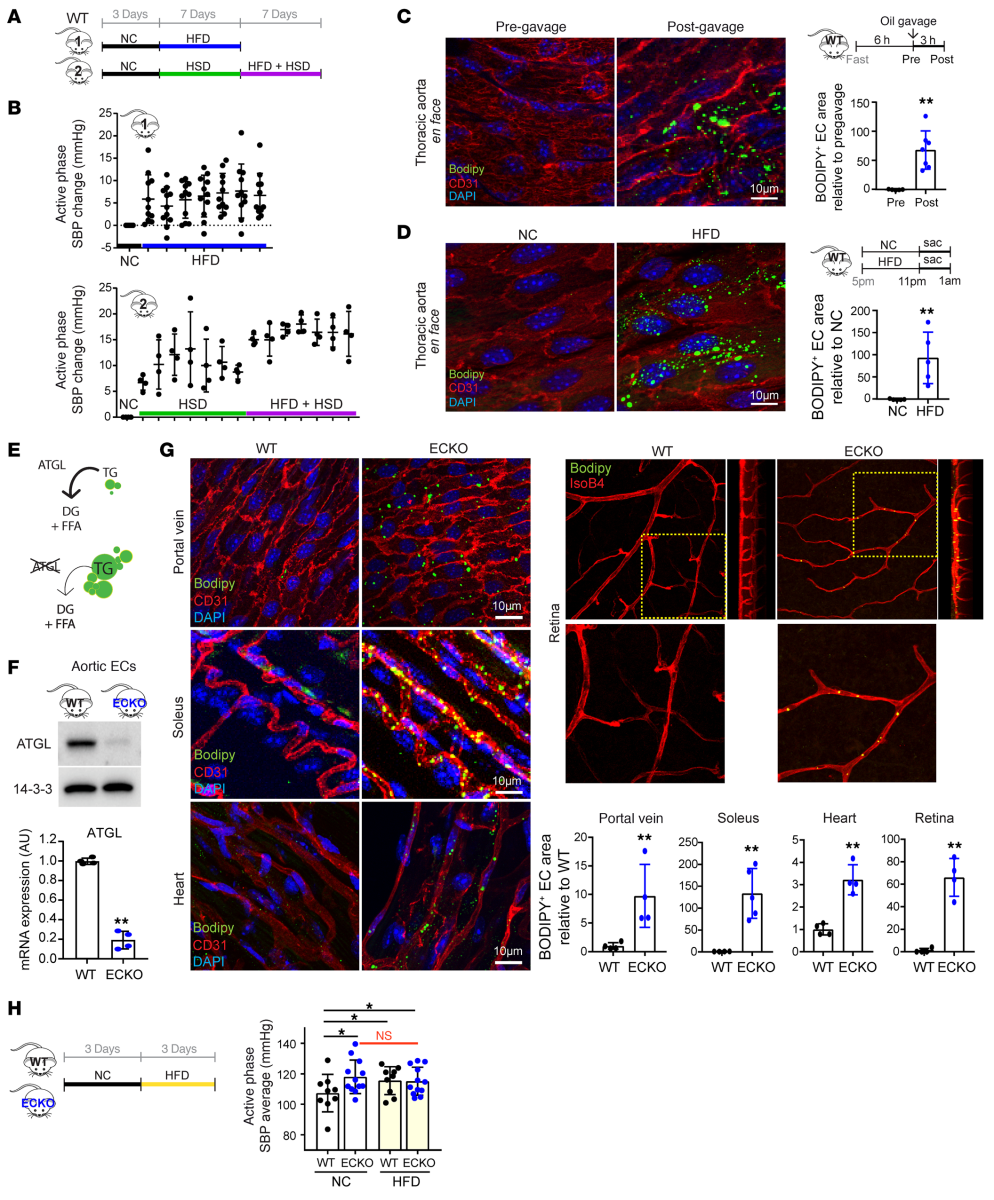
Endothelial deletion of *Atgl* phenocopies fat intake–induced accumulation of LDs and rise in BP. (**A**) Experimental setup for administration of NC, HFD, HSD, or HFD+HSD in WT C57BL/6J mice, while monitoring BP by noninvasive telemetry. (**B**) Elevation of SBP during active phase (7 pm to 7 am) under indicated diet for 7 days. *n* = 11 (group 1); *n* = 4 (group 2). One-way ANOVA**.** (**C** and **D**) En face staining of thoracic aorta before and after olive oil gavage (10 mL/kg body weight) (**C**) or 5 hours of either NC or HFD ad libitum feeding (**D**) in WT C57BL/6J mice. BODIPY staining (green) indicates neutral lipids, and CD31 (red) marks the endothelium. BODIPY-positive area in the endothelium is quantified (right panel). *n* = 4–7 (**C**); *n* = 5 (**D**). **P < 0.01, *t* test. (**E**) Schematic of the role of ATGL in TG hydrolysis, yielding diacylglycerols (DG) and FFA. Deletion of ATGL leads to LD accumulation. (**F**) WB (upper panel) and qPCR (lower panel) of isolated aortic ECs from WT versus *Atgl* ECKO mice. *n* = 4. **P < 0.01, *t* test. (**G**) Whole-mount staining of portal vein, soleus, heart, and retina from fasted WT versus *Atgl* ECKO mice, imaged with BODIPY (green), anti-CD31 immunohistochemistry or IsoB4 lectin (red), and DAPI (blue). For the retina staining, side views of *Z*-stacked images are shown on the right, and zoomed-in images are shown below. BODIPY-positive area in the endothelium is quantified (right panel). *n* = 4–5. **P < 0.01, *t* test. (**H**) Left panel: experimental setup for administration of NC or HFD in WT versus *Atgl* ECKO mice. Right panel: average active-phase SBP in each genotype while provided with the indicated diet. *n* = 9 (WT); *n* = 12 (*Atgl* ECKO). *P < 0.05, 1-way ANOVA.

To study the impact of fat intake on BP, WT C57BL/6J mice were assigned to receive normal chow (NC), a high-fat diet (HFD), a high-salt diet (HSD), or both HFD+HSD, as outlined in Figure 1A, and BP was continually evaluated with an implantable telemetry monitoring system that recorded BP every 5 minutes. HSD diet has been widely shown to elevate BP in mice (21, 22) and was included here both as positive control and to determine whether interaction with HFD occurs. The use of telemetry permits measurements of BP in unrestrained mice throughout the day and, more importantly, night (active phase in mice), and without operator interference — important benefits over other options such as the tail-cuff system. As shown in Figure 1B and Supplemental Figure 1, the addition of HFD increased systolic BP (SBP) (Figure 1B and Supplemental Figure 1B; supplemental material available online with this article; https://doi.org/10.1172/JCI173160DS1) and mean BP (Supplemental Figure 1D) in mice by 5 to 10 mmHg independently of the coadministration of HSD and in the active phases of the day. No significant differences were noted in BP measurements during the inactive phase (Supplemental Figure 1, E–G).

Kuo et al. have recently shown that ECs induce the storage of fats into LDs upon exposure of excessive amounts of FAs (20). Their work demonstrated, and our data fully reproduce, that oleic acid (OA) induces LD formation in HUVECs (Supplemental Figure 2) and that administration of olive oil oral gavage to mice causes accumulation of LDs in the thoracic endothelium (Figure 1C). To investigate whether physiological fat intake could also promote LD formation, we examined the thoracic endothelium after 6 hours of ad libitum consumption of HFD. We observed a significant induction of LDs, similar to that seen after olive oil gavage (Figure 1D), which persisted and became more pronounced after 4 weeks of HFD (Supplemental Figure 3). Moreover, evaluation of portal vein (Supplemental Figure 4A) and capillaries of skeletal muscle (Supplemental Figure 4B) demonstrated that LD accumulation also occurred in vascular beds beyond the thoracic endothelium, including the microvasculature. We conclude that the elevation of BP in animals fed a HFD is accompanied by accumulation of LDs throughout the vasculature.

### Endothelial LDs are sufficient to elevate BP in vivo.

Based on the observations above and the fact that endothelium is crucial in regulating BP, we hypothesized that endothelial LD accumulation mediates fat intake–induced BP elevation. To test this hypothesis, we generated mice that lack *Atgl* specifically in the endothelium (*Atgl* ECKO mice: *Atgl^fl/fl^*;Vecad-Cre). ATGL is required for lipolysis of LDs (23) (schematics in Figure 1E), and inhibition of ATGL in cell culture and in ex vivo aorta culture causes accumulation of LDs in ECs (Supplemental Figure 5), as shown previously (20). Isolated ECs from *Atgl* ECKO mice revealed an approximately 90% reduction in ATGL protein and transcript (Figure 1F), indicating efficient KO of *Atgl* in ECs. Consistent with findings in cell culture, the *Atgl* ECKO mice revealed LD accumulation in endothelium of numerous tissues, including heart, skeletal muscle, portal vein, and retina (Figure 1G). These mice thus afford the opportunity to test the systemic impact of endothelial LD accumulation in the absence of the other numerous effects of HFD. *Atgl* ECKO mice were born in Mendelian ratios and appeared grossly normal with normal vasculature development (Supplemental Figure 6), body weight (Supplemental Figure 7A), body composition (Supplemental Figure 7B), plasma lipid profiles (Supplemental Figure 7C), oral fat tolerance (Supplemental Figure 7D), glucose tolerance (Supplemental Figure 7E), and plasma insulin levels whether fasted, fed, or provided a HFD (Supplemental Figure 7F). We found, however, that BP in *Atgl* ECKO mice was higher by 5 to 10 mmHg compared with that in littermate control animals (Figure 1H). We conclude that LD accumulation in ECs is sufficient to facilitate BP elevation in vivo. Moreover, the BP elevation in NC-fed *Atgl* ECKO mice was identical to that seen in control animals in response to HFD, and the provision of HFD to *Atgl* ECKO mice had no further impact on BP (Figure 1H), consistent with the hypothesis that HFD-induced elevation of BP is mediated by LD accumulation in the endothelium, i.e., that the *Atgl* ECKO mice model the effect of HFD in the endothelium. In contrast, BP in *Atgl* ECKO mice was increased by an additional 5 to 10 mmHg in response to HSD challenge compared with littermate control animals (Supplemental Figure 8), indicating that HSD induces BP by a different mechanism.

### Endothelial LDs suppress eNOS and vasodilation.

**Figure 2:**
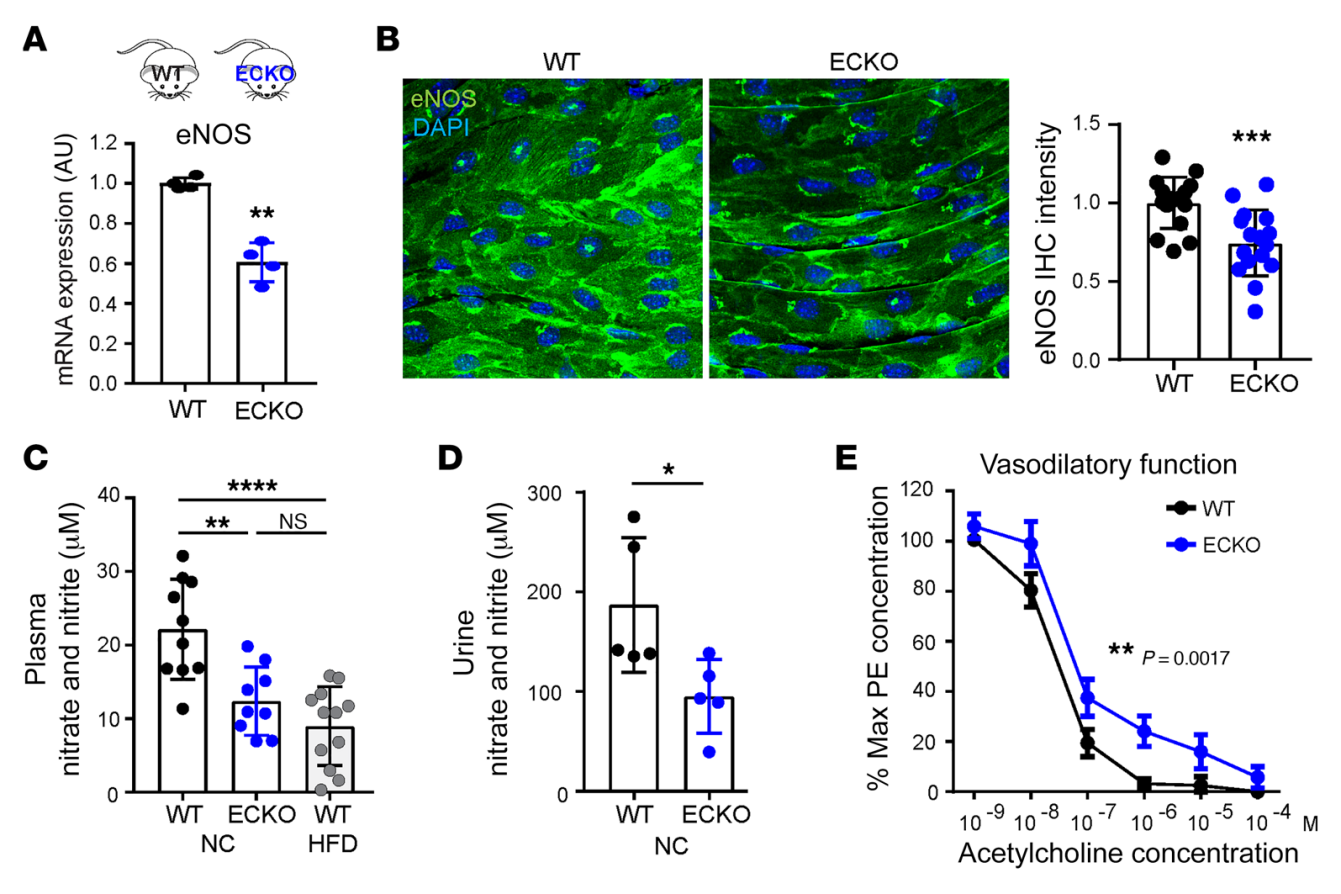
Endothelial deletion of ATGL suppresses eNOS and vasodilation. (**A**) qPCR quantification of eNOS mRNA in ECs isolated from the lung of WT versus *Atgl* ECKO mice. *n* = 4. **P < 0.01, *t* test. (**B**) En face staining of eNOS protein in thoracic aorta from WT versus *Atgl* ECKO mice. Quantification of eNOS (green) fluorescence intensity (right panel). *n* = 16. ***P < 0.001, *t* test. Images were captured using a ×40 lens with a ×2 digital zoom. (**C**) Nitrate and nitrite levels measured in plasma from WT versus *Atgl* ECKO mice receiving NC and WT mice receiving HFD for 6 weeks. *n* = 9–12. **P < 0.01; ****P < 0.0001, 1-way ANOVA. *n* = 9–12 mice/group. (**D**) Nitrate and nitrite levels measured in urine from WT versus *Atgl* ECKO mice. *P < 0.05, *t* test. *n* = 5 mice/group. (**E**) Quantification by pressure myography of the vasodilatory response to ACh by carotid arteries explanted from WT versus *Atgl* ECKO mice. **P < 0.01, paired *t* test. *n* = 6 mice/group.

**Figure 3:**
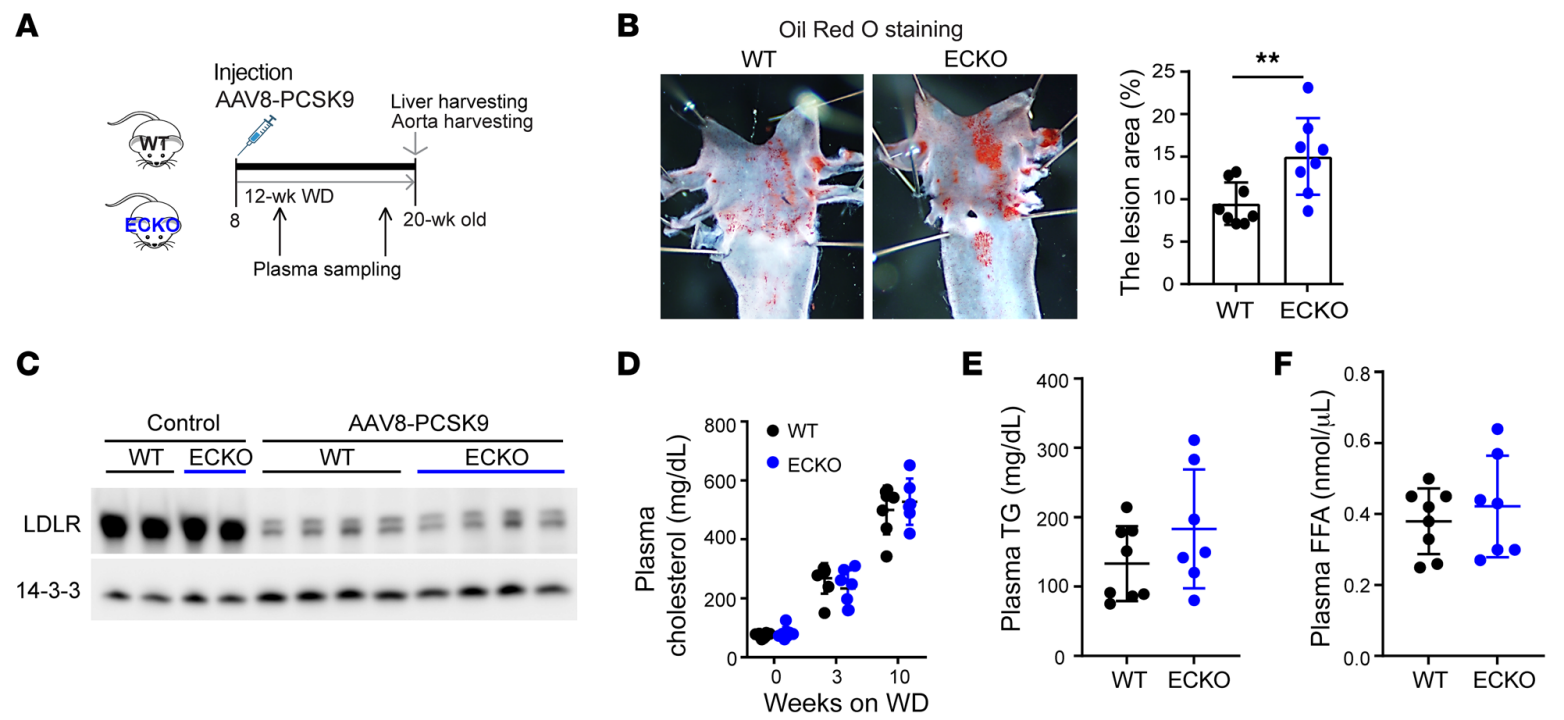
Endothelial deletion of ATGL accelerates atherosclerosis in the PCSK9 overexpression model. (**A**) Schematic of experimental setup for AAV8-PCSK9 injection–induced atherosclerosis model. WD, Western diet. (**B**) Oil Red O staining of aortic arch in WT versus *Atgl* ECKO mice and quantification of Oil Red O–positive lesion area. **P < 0.01, *t* test. *n* = 8 mice/group. (**C**) LDLR protein levels in the liver of WT versus *Atgl* ECKO mice with or without AAV8-PCSK9 injection. (**D**–**F**) Plasma cholesterol, TG, and FFA measurements in WT versus *Atgl* ECKO mice at the indicated time points following AAV8-PCSK9 injection. *n* = 7–8.

NO, generated by eNOS, is a dominant endogenous vasodilator and regulator of BP in vivo. Strikingly, expression of eNOS was markedly suppressed in the ECs of *Atgl* ECKO mice, as seen in isolated aortic ECs (~40%, Figure 2A) and by aorta en face staining (~35%, Figure 2B). Consistent with this reduced eNOS expression, *Atgl* ECKO mice had substantially fewer NO byproducts, nitrates and nitrites, in the plasma (Figure 2C) and urine (Figure 2D). Notably, the reduction of plasma NO byproducts in *Atgl* ECKO mice was comparable to that seen in mice fed a HFD (Figure 2C). The observed reduction in NO byproducts induced by HFD is likely to be mediated through the suppression of eNOS, as this effect was abolished in eNOS-KO mice (Supplemental Figure 9). Consistent with reduced NO production, carotid arteries isolated from *Atgl* ECKO mice, compared with those of littermate controls, revealed impaired endothelium-dependent vasodilatory function at physiological pressure, as determined by pressure myography (Figure 2E). Also consistent with impaired NO production, which is known to promote atherosclerosis (24, 25), *Atgl* ECKO mice were predisposed to atherosclerosis in the AAV8-PCSK9 (proprotein convertase subtilisin/kexin type 9) model (Figure 3, A and B), despite equivalent reduction in liver expression of LDLR protein (Figure 3C) and hyperlipidemia (Figure 3, D–F) as in control mice.

**Figure 4:**
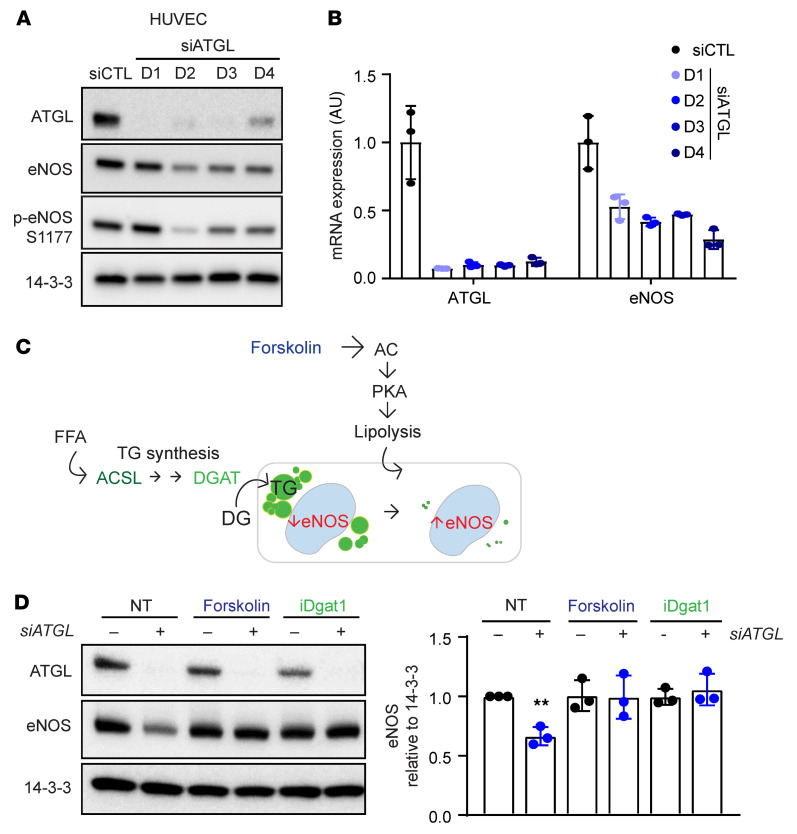
Endothelial knockdown of ATGL suppresses eNOS via the accumulation of LDs. (**A** and **B**) WB of the indicated proteins (**A**) and quantification by qPCR of the indicated mRNAs (**B**) days 1–4 (D1–D4) after knockdown of ATGL by siRNA transfection in HUVECs. *n* = 3. (**C**) Schematic indicating the 2 approaches taken to reducing LD burden: enhancing lipolysis (with forskolin) or blocking TG synthesis (with siACSL or siDGAT1, see Supplemental Figure 10; or with DGAT inhibition). (**D**) WB of ATGL and eNOS in HUVECs treated for 2 days with siATGL, forskolin, or iDGAT1, as indicated. Quantification of eNOS protein levels relative to 14-3-3 is shown in right panel. *n* = 3. **P < 0.01, 1-way ANOVA.

To determine whether the suppression of eNOS seen with loss of ATGL was cell autonomous, we turned to cell culture studies using HUVECs. Silencing of ATGL with siRNA led to rapid suppression of eNOS protein (~50%, Figure 4A and Supplemental Figure 10A) and transcript (>50%, Figure 4B) within 24 hours in both HUVECs and primary cultured mouse aortic ECs. There was no evidence of decreased eNOS activation, as measured by the ratio of phosphorylated eNOS (p-eNOS)/eNOS (Figure 4A), or of decreased eNOS protein stability (Supplemental Figure 10B). Treating cells with a series of PPARα and PPARγ agonists did not rescue eNOS mRNA (Supplemental Figure 10C), indicating that the suppression of eNOS is not mediated by sequestration of PPAR ligands in LDs of cells lacking ATGL. The data are thus most consistent with eNOS inhibition being mediated by LDs themselves. To formally test this notion, we sought to reduce the LD burden in cells either by enhancing lipolysis with forskolin, which activates adenylate cyclase to promote lipolysis, or by preventing LD formation by pharmacologic or genetic suppression of DGAT1 or ACSL1, enzymes required for TG synthesis (Figure 4C). In all cases, LD burden in ECs was nearly abrogated (Supplemental Figure 10C) and eNOS was entirely rescued (Figure 4D and Supplemental Figure 10E). Inhibition of FA oxidation (FAO) did not block the rescue of eNOS by activation of lipolysis (Supplemental Figure 10F), indicating that the suppression of eNOS by LDs is not mediated by sequestration of substrates for FAO. Together, these data indicate that the accumulation of LDs in ECs suppresses eNOS mRNA levels and NO production in a cell-autonomous fashion in cell culture and in vivo, leading to impaired endothelium-dependent vasorelaxation.

### Endothelial LD accumulation leads to eNOS mRNA destabilization.

**Figure 5:**
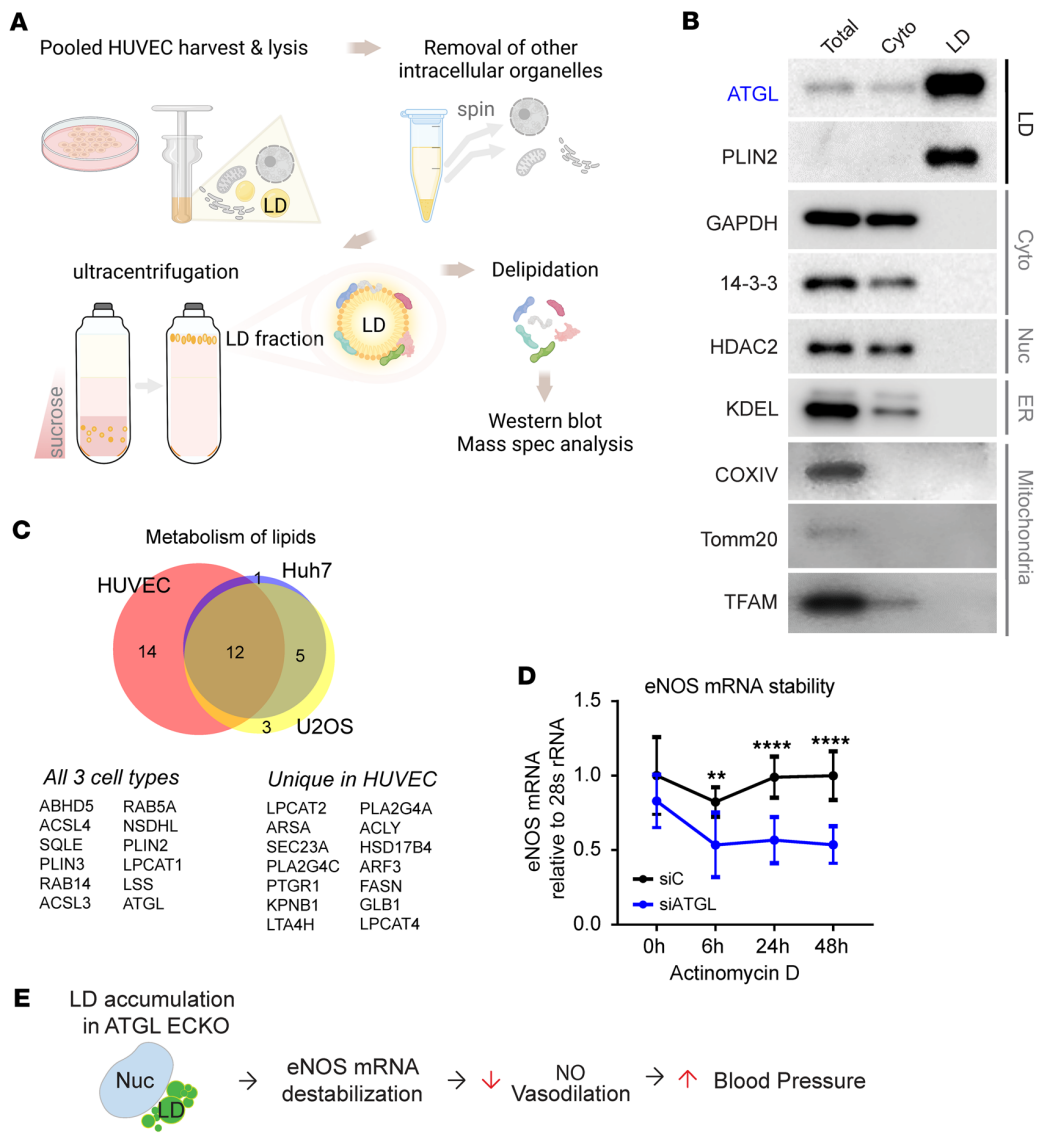
Endothelial LD accumulation leads to eNOS mRNA destabilization. (**A**) Schematic of LD purification experiment in HUVECs. Spec, spectrometry. (**B**) WB of indicated intracellular organellar marker proteins in total, cytosolic, and LD fraction. (**C**) Venn diagram comparing the LD proteomics data sets of HUVECs and Huh7 and U2OS cells involved in lipid metabolism. List of overlapping in all 3 cell types and unique LD proteins in HUVECs. (**D**) eNOS mRNA stability measurements in siCTL versus siATGL HUVECs in response to actinomycin D treatment (5 nM). eNOS mRNA levels at indicated time points following actinomycin D were normalized to 28s rRNA. *n* = 8. **P < 0.01; ****P < 0.0001, 2-way ANOVA. (**E**) Model showing LD accumulation suppresses eNOS mRNA stability, NO production, and vasodilatory capacity, leading to BP elevation.

To further investigate the mechanism by which endothelial LDs suppress eNOS transcript levels, we conducted unbiased proteomics analyses, using mass spectrometry (MS), on highly purified LDs from HUVECs (Figure 5, A and B). The identified LD-associated proteins (Supplemental Table 1) overlapped substantially with those identified in previously published LD proteomics data sets from other cell types (26), particularly including proteins associated with lipid metabolism (Figure 5C and Supplemental Table 2). There were, however, also numerous proteins uniquely identified in the endothelial LD-associated proteome. Gene Ontology (GO) analysis of the identified endothelial LD proteome revealed a highly significant overrepresentation of proteins involved in the regulation of mRNA stability (GO 0043488) (Supplemental Table 3). These findings suggested that LDs may affect the stability of eNOS mRNA. Consistent with this notion, treating cells with actinomycin D to suppress active transcription revealed accelerated degradation of eNOS mRNA in ATGL-silenced ECs, compared with control cells (Figure 5D). Three proteins are known to regulate eNOS mRNA stability, EEF1A1 (27), PTBP1 (28), and PCBP1 (29). Strikingly, all 3 of these proteins were identified as associated with LDs in our proteomics data set (Supplemental Table 1). siRNA knockdown of one of these, PCBP1, fully rescued eNOS mRNA levels (Supplemental Figure 11A) and mRNA stability (Supplemental Figure 11B) at 48 hours, although interestingly, not at 96 hours (Supplemental Figure 11C). The LD localization of PCBP1 protein was confirmed with immunostaining in siATGL ECs (Supplemental Figure 11D). Together, these data delineate a model in which the accumulation of LDs destabilizes eNOS mRNA, ultimately leading to reduced NO production, impaired vasodilatory capacity, and a propensity to elevations in BP (Figure 5E).

### Endothelial LDs also activate NF-κB and induce MCP1.

**Figure 6:**
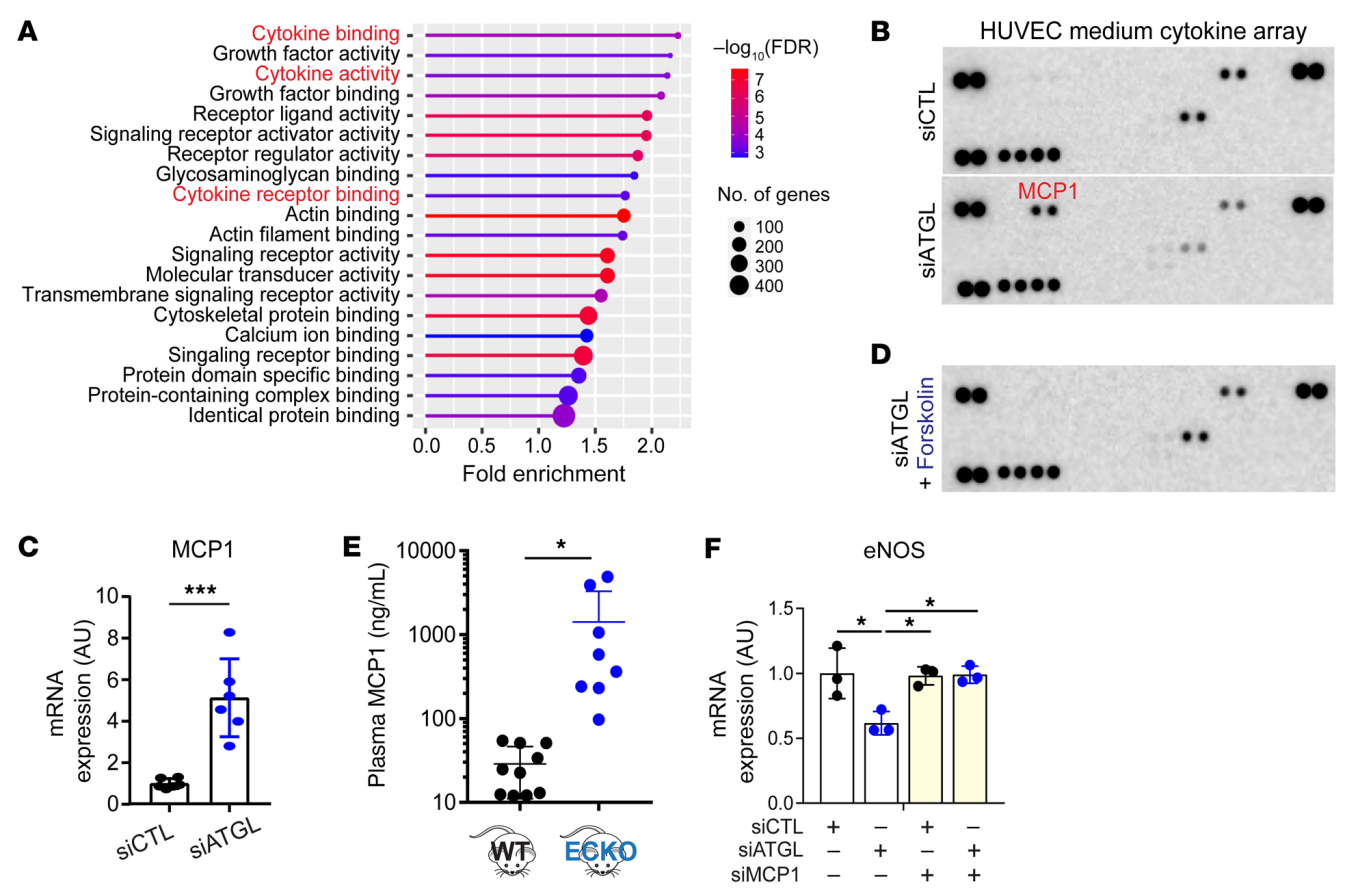
Endothelial LDs induce MCP1 production. (**A**) Molecular function GO analysis of differentially expressed genes in HUVECs treated with siCTL versus siATGL. (**B**) Cytokine array assay with media conditioned by HUVECs treated with siCTL versus siATGL. (**C**) Quantification of MCP1 mRNA by qPCR in HUVECs treated with siATGL. *n* = 6. ***P < 0.001, *t* test. (**D**) As in **B**, simultaneously treated with forskolin. (**E**) Luminex analysis of MCP1 levels in plasma from WT versus *Atgl* ECKO mice. *P < 0.05, *t* test. *n* = 8–10 mice. (**F**) Quantification of eNOS mRNA by qPCR in HUVECs treated with the indicated siRNAs. *n* = 3. *P < 0.05, 1-way ANOVA.

To identify additional mechanisms by which LDs suppress eNOS expression, we performed RNA-Seq analysis in HUVECs after silencing of ATGL versus control cells. ShinyGO, version 0.741 (30), molecular function GO analysis of the results indicated a broad activation of cytokine pathways in ATGL-silenced cells (Figure 6A). We therefore quantified cytokines secreted by siATGL and control HUVECs in cell culture. MCP1 (also known as CCL2) was uniquely and potently induced by silencing of ATGL (Figure 6B), reflecting its increased expression noted by quantitative PCR (qPCR) (Figure 6C). As with eNOS expression, treating cells with forskolin normalized MCP1 expression (Supplemental Figure 12A) and protein secretion (Figure 6D) in siATGL HUVECs, indicating that LDs were responsible for the induction of MCP1. Consistent with the cell culture data, a dramatic induction of MCP1 was observed in plasma from *Atgl* ECKO animals compared with controls (Figure 6E and Supplemental Figure 12B). Strikingly, silencing of MCP1 rescued eNOS expression in siATGL cells (Figure 6F), indicating that LDs suppress eNOS expression at least in part via MCP1. The transcription factor complex NF-κB is known to promote the expression of proinflammatory cytokines, including MCP1 (31), and its increased activity has been implicated in endothelial dysfunction, including the suppression of eNOS expression (32, 33). Silencing of NF-κB in cells lacking ATGL prevented the induction of MCP1 (Supplemental Figure 12C) and rescued the suppression of eNOS (Supplemental Figure 12D), indicating that the suppression of eNOS and induction of MCP1 by LDs is mediated by NF-κB. Together, these data suggest that, in addition to eNOS mRNA destabilization, the accumulation of LDs also activates a proinflammatory pathway of the NF-κB/MCP1 axis.

### Normalizing endothelial LDs restores BP.

**Figure 7:**
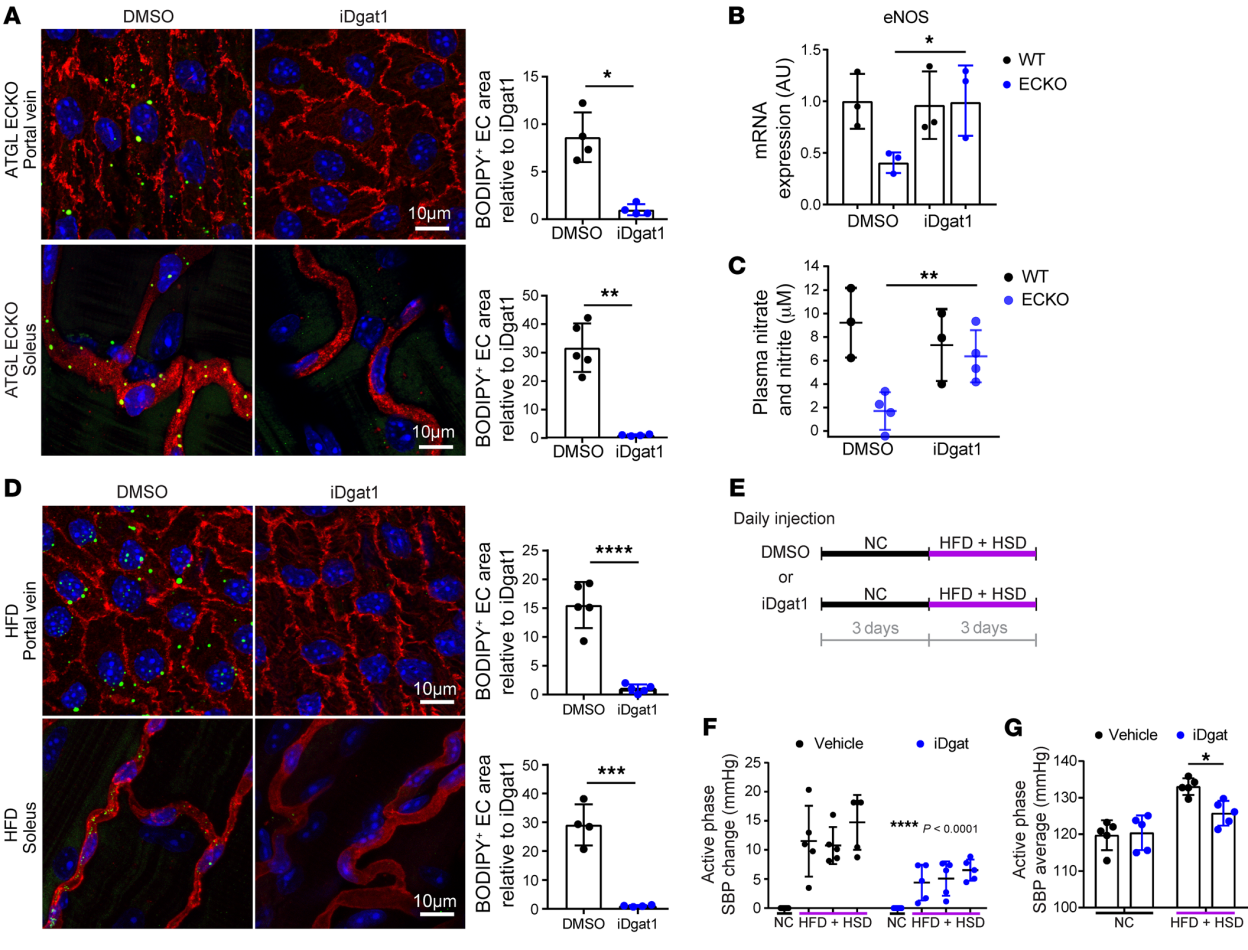
Suppression of LD formation rescues the induction of BP by endothelial ATGL deletion or by HFD. (**A**) En face staining of portal vein (upper panel) and whole-mount staining of capillary vessels in the soleus (lower panel) after DMSO versus iDGAT1 injection in *Atgl* ECKO mice. iDGAT1 was given at 3 mg/kg via i.p. injection. BODIPY staining (green) indicates neutral lipids, and CD31 (red) marks the endothelium. BODIPY-positive area in the endothelium is quantified. *P < 0.05; **P < 0.01, *t* test. *n* = 4–5 mice/group. (**B**) eNOS mRNA levels measured in isolated ECs from lung of WT versus *Atgl* ECKO mice after a week of DMSO versus iDGAT1 injection. *n* = 3. *P < 0.05, 1-way ANOVA. (**C**) Nitrate and nitrite levels measured in the plasma of WT versus *Atgl* ECKO mice after a week of DMSO or iDGAT1 injection (3 mg/kg i.p.). **P < 0.01, 1-way ANOVA. *n* = 3–4 mice/group. (**D**) En face staining of portal vein (upper panel) and whole-mount staining of capillary vessels in the soleus (lower panel) after DMSO versus iDGAT1 injection (3 mg/kg i.p.) in C57BL/6J WT mice maintained on a 3-day HFD. BODIPY staining (green) indicates neutral lipids, and CD31 (red) marks the endothelium. BODIPY-positive area in the endothelium is quantified on the right. *n* = 4–5. ***P < 0.001; ****P < 0.0001, *t* test. (**E**) Experimental setup of daily administration of DMSO or iDGAT1 in C57BL/6J WT mice while providing NC or HFD+HSD. (**F**) Elevation of SBP during the active phase while provided with the indicated diet. ****P < 0.0001, 2-way ANOVA. *n* = 5 mice/group. (**G**) Average active phase SBP while provided with the indicated diet. *P < 0.05, 1-way ANOVA. *n* = 5 mice/group.

To investigate the role of LDs in the regulation of NO and BP in vivo, we treated mice with the DGAT inhibitor iDGAT1 (34) to suppress LD formation. Treatment of *Atgl* ECKO mice with iDGAT1 suppressed LD accumulation in the endothelium of both large and small vessels (Figure 7A), demonstrating the requirement of DGAT for LD formation in ECs in vivo. Although no suppression of hyperlipidemia was observed (plasma FFAs and TGs, Supplemental Figure 13) with iDGAT1 treatment, we observed the restoration of eNOS mRNA (Figure 7B) and NO production in *Atgl* ECKO mice, as evidenced by nitrate and nitrite levels in the plasma (Figure 7C). These findings demonstrate that endothelial LDs inhibit NO production in vivo. In parallel, treatment of HFD-fed mice with iDGAT1 also suppressed LD accumulation in the endothelium of both large and small vessels (Figure 7D), demonstrating that LD formation in ECs after physiological lipid loading also requires DGAT. Finally, treatment with iDGAT1 reduced diet-induced BP elevation by approximately 50% (Figure 7, E–G), demonstrating that LDs promote BP elevation in vivo.

### Expression of LD-associated genes and BP in humans.

**Figure 8:**
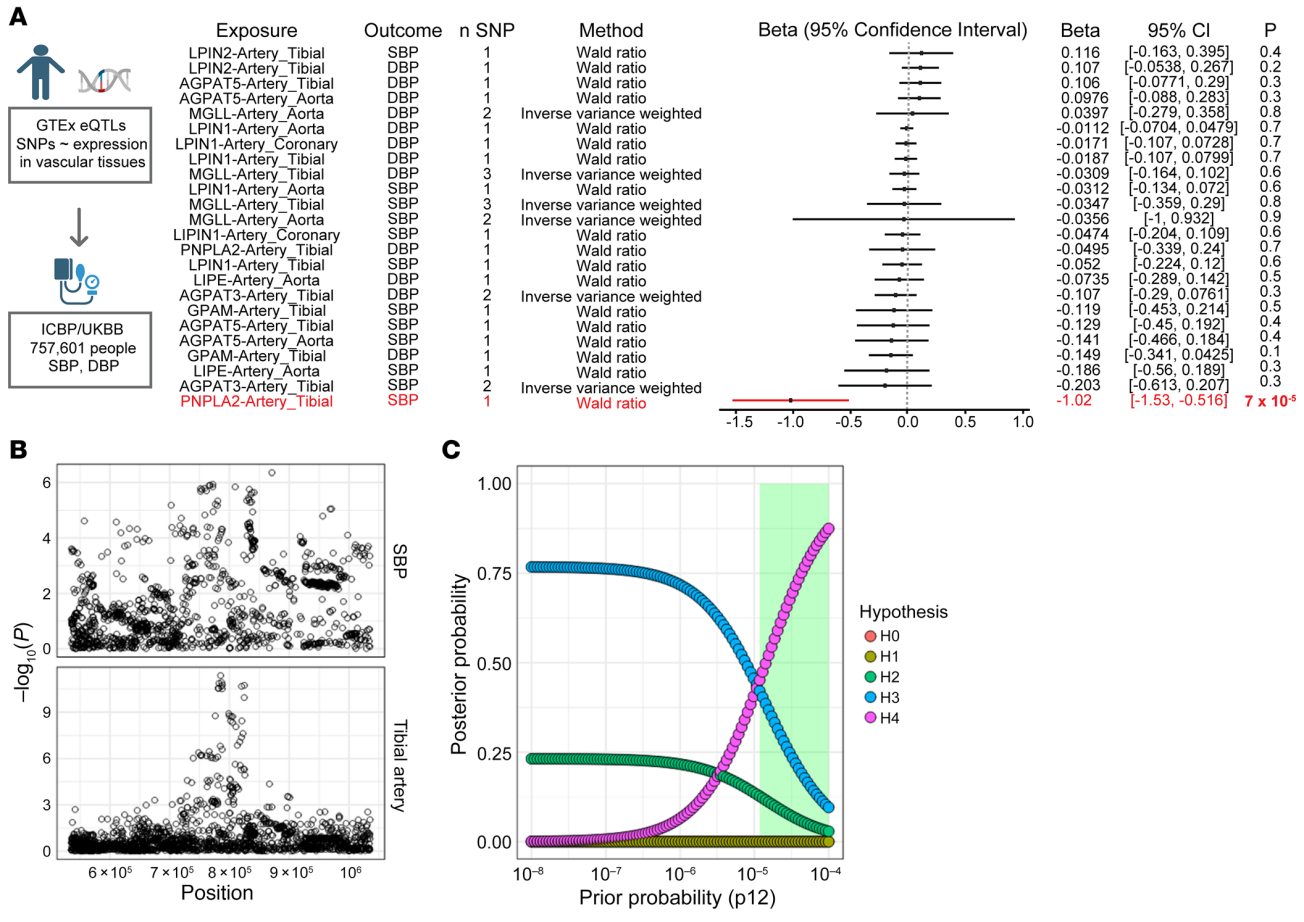
Association of vascular ATGL expression with BP in humans. (**A**) Results of 2-sample inverse variance weighted Mendelian randomization, testing the effects of expression of LD-associated genes in vascular tissues (aorta, coronary artery, tibial artery) on SBP and DBP. (**B**) Regional association plots highlighting ± 250 kb surrounding the *PNPLA2* locus for SBP (top) and tibial artery (bottom) on chromosome 11. (**C**) Results of Bayesian enumeration colocalization sensitivity analysis. Each hypothesis corresponds to a different causal configuration, and the posterior probability of each hypothesis is plotted across a range of prior probabilities (default *P*_12_ = 1 × 10^–5^). H0, neither trait has a genetic association in the region; H1, only SBP has a genetic association in the region; H2, only *PNPLA2* expression in tibial artery has a genetic association in the region; H3, both traits have genetic associations, but different causal variants; and H4, both traits have genetic associations and share a single causal variant. From the default (default *P*_12_ = 1 × 10^–5^) to more optimistic (*P*_12_ = 1 × 10^–4^) priors (corresponding to approximately 1.3% and 13% probabilities of a shared causal variant), there is intermediate (41.2%) to strong (87.5%) posterior probability for a shared causal variant at the *PNPLA2* locus surrounding the lead eQTL. The shaded green region denotes the range of prior probabilities which lead to H4 > H3.

Finally, to investigate the role of endothelial LD accumulation in human BP control, we turned to Mendelian randomization studies. We constructed genetic instruments that proxy changes in vascular LD content, using genetic variants associated with the expression of genes involved in LD formation or breakdown (*PNPLA2*, *GPAM*, *AGPAT1*, *AGPAT3*, *AGPAT5*, *LPIN1*, *LPIN2*, *DGAT1*, *DGAT2*, *LIPE*, and *MGLL*) in vascular tissues (aorta, coronary artery, and tibial artery) from GTEx, version 8 (35, 36). Application of these instruments to the UK Biobank (UKB) (https://www.ukbiobank.ac.uk/) revealed that increased expression of *PNPLA2* (which encodes ATGL) was associated with decreased SBP (β = –1.02 mmHg/1 unit increase in normalized effect size, 95% CI, –1.53 to –0.52, *P* = 7 × 10^–5^) (Figure 8A). We did not detect associations between expression of other LD-related genes and SBP or diastolic BP (DBP), although wide confidence intervals limit our ability to exclude meaningful effects of these other genes on BP. In a sensitivity analysis, we performed Bayesian enumeration colocalization to evaluate the presence of a shared causal variant influencing both expression of PNPLA2 and SBP (37), finding intermediate-to-strong evidence in support of a shared causal variant (Figure 8, B and C). We conclude that vascular expression of *PNPLA2* in humans correlates inversely with SBP, consistent with the notion that LD accumulation predisposes to BP elevation.

## Discussion

Elevations in BP are widely associated with obesity as part of the metabolic syndrome. Endothelial dysfunction and inflammation have been implicated in this association, but how endothelial dysfunction is triggered remains unclear (38, 39). Our study provides a partial mechanistic explanation for this association: excess lipids drive the accumulation of LDs in the endothelium, in turn suppressing NO production and endothelium-dependent vasodilation. BP elevation is multifactorial, and other mechanisms certainly exist, including activated renin/angiotensin system, systemic inflammation, and sympathetic overactivity (39, 40). Some of these mechanisms likely synergize. For example, we find that LDs in the endothelium promote an inflammatory response, which may well synergize with a systemic inflammatory response triggered by metabolically unhealthy adipose tissue in obesity. Evaluating such potential interactions will be of future interest.

The study of LD biology has recently resurged, with an appreciation for their role beyond energy storage in multiple cell types (17–19). We show here that LDs also play an active role in vascular pathobiology, contributing to elevated BP under lipid load. This process may also play a physiologic role: we find that LDs accumulate in the endothelium under physiologic postprandial conditions, and we have suggested previously that the accumulation of LDs in the endothelium may represent a mechanism to protect underlying parenchyma from lipotoxicity (41). Our current findings add to this paradigm: suppression of vasodilation by LDs may serve a similar purpose via reduction of local blood flow and thus excess nutrient delivery.

We identify here a mechanism by which lipid loading and high-fat intake acutely promote elevation in BP within 1 to 2 days of HFD consumption. HFD affects body weight and insulin resistance over weeks only (42), thus indicating that the acute effects are independent of effects on insulin resistance. On the other hand, the relationship between these acute events and obesity per se is likely complex, as is the interpretation of the multifactorial impact of chronic diet studies. For example, ketogenic diets, which have high fat content, might be predicted to elevate BP, but the effect is complicated by the fact that ketones themselves have a countering vasodilatory effect and that these diets are quite effective at promoting weight loss, thereby also promoting reductions in BP. Nevertheless, for example, Guo et al. (43) found that ketogenic diet increased BP by 20 mmHg in spontaneously hypertensive rats, impaired vasodilation, suppressed endothelial eNOS and NO production, and activated endothelial NF-κB, which is remarkably similar to our findings in HFD mice. A metaanalysis of ketogenic diets in humans showed little effect on SBP and a trend to lowering DBP that was only apparent in studies with 24 months of follow-up, concordant with weight loss (44).

Our Mendelian randomization results strongly support the relevance of our findings in human populations. The relationship between *PNPLA2* (*ATGL*) expression and BP was seen only with SBP, but this may well be a methodological limitation. The determinants of SBP are quite complex and include peripheral resistance, stroke volume, duration of systole, cardiac contractility, and vascular impedance (i.e., stiffness of conduit arteries). The same can be said for DBP, including peripheral resistance, duration of diastole (and thus heart rate), and vascular elastance and compliance. Moreover, cuff measurements of SBP and DBP are variable and incompletely correlative to intraarterial pressures (45).

How do LDs in the endothelium suppress eNOS? We identify 2 potential mechanisms: suppression of eNOS mRNA stability and activation of an inflammatory cascade, dominated by MCP1. Rescue experiments conclusively implicate both pathways: knockdown of PCBP1 and of MCP1 independently rescued mRNA expression of eNOS. However, how these two processes relate to each other, if at all, is not clear. We have, for example, not identified conclusive evidence indicating that the NF-κB/MCP1 axis regulates mRNA stability. The two pathways may thus function independently. Precisely how LD accumulation activates the NF-κB pathway is also not clear at this time; our proteomic studies of LD-associated proteins did not provide obvious hypotheses. In contrast, the LD-associated proteomics data set strikingly contained all 3 proteins reported to regulate eNOS mRNA stability: EEF1A1 (27), PTBP1 (28), and PCBP1 (29). Exactly how LDs communicate to these proteins remains unclear. LDs are generated by, and communicate with, the endoplasmic reticulum (ER), and PCBP1 has been reported at the ER (46), suggesting that some of these LD-associated proteins may reflect LD-ER interactions. The rescue of eNOS mRNA stability by knockdown of PCBP1 was somewhat surprising, as PCBP1 is reported as a stabilizer of eNOS mRNA, not a destabilizer. On the other hand, we also find that knockdown of PCBP1 does suppress eNOS mRNA over a longer period of time, indicating the presence of important feedback loops. These intricate regulatory mechanisms will warrant thorough exploration in future studies.

In summary, our results unveil LDs in the endothelium as bioactive hubs with important effects on vascular function, providing an important link between the metabolic syndrome and BP.

## Methods

### Atgl ECKO mice generation.

*Atgl^fl/fl^* mice were obtained from Erin Kershaw (University of Pittsburgh, Pittsburgh, Pennsylvania, USA) (47) and bred with transgenic mice expressing Cre recombinase under the VE-cadherin promoter provided by Nancy Speck (University of Pennsylvania).

### BP measurement using radio telemetry.

Continuous 24-hour SBP and DBP were monitored in unrestrained mice by using implantable HD-X10 telemetry (Data Science, DSI). Eight- to ten-week-old C57BL/6J WT male mice were used for the experiments presented in Figure 1, A and B, and Supplemental Figure 1. Briefly, after recovering from implantation surgery, mice were kept under a 12-hour light/12-hour dark cycle and fed a NC diet. During that time, baseline SBP and DBP were measured every 5 minutes for 3 days. Then the mice were fed with either HFD (60% kcal% fat; Research Diet, D12492) or HSD (8% NaCl diet; Envigo, TD92012) and their BPs were again measured continuously for 7 days. Then both groups of mice were fed with HFD+HSD (60 kcal% fat and 8% added NaCl; Research Diet, D12060102). Data are expressed as daily change in BP and as average BP with each diet. For the experiments presented in Figure 1H and Supplemental Figure 8, WT versus *Atgl* ECKO male mice at 12 weeks of age were used. After recovering from telemetry implantation surgery, both groups of mice were fed with NC for 3 days and then with HFD or HSD for 3 days. Data are expressed as daily change in SBP and as average SBP with each diet. For the experiments presented in Figure 7, F and G, 12-week-old C57BL/6J WT male mice were used. After recovering from implantation surgery, mice were randomly assigned to either the DMSO or iDGAT1 injection group. All mice were first fed with NC for 3 days and then switched to HFD+HSD for 3 days. Throughout the study, the mice were i.p. injected with the assigned drug daily (in between 8 am and 9 am) at 3 mg/kg. Data are expressed as daily change in SBP from 5 mice per group and as average SBP with each diet.

### Lipid administration to mice.

For gavage studies, mice were fasted for 6 hours and then administered oral gavage of olive oil (10 mL/kg body weight). Blood vessels were collected 3 hours after gavage for en face staining to visualize LDs in the endothelium in both male and female mice. For the oral fat–tolerance test, tail blood was collected at the indicated time points for TG assay in male mice. For the ad libitum HFD study, mice were fasted for 6 hours and were given HFD for 6 hours during their active phase, from 5 pm to 11 pm. The vessels were harvested after 11 pm for en face staining to visualize LDs in the endothelium in both male and female mice.

### Mouse tissue immunohistochemistry.

For en face staining of the large vessels or whole-mount staining of capillaries in the skeletal muscle or heart, mice were perfused with cold PBS and then with 3.7% PFA. The vessels or tissues were dissected and were further fixed in 3.7% PFA for another hour. Then, the tissues were washed, permeabilized with 0.3% Triton X-100, blocked with 3% BSA, and incubated with anti-mouse CD31 antibody (MilliporeSigma, MAB1398Z) overnight at 4°C. The next day, tissues were washed and incubated with secondary antibody (anti-hamster AF594) (Jackson ImmunoResearch, 127-585-160) for 2 hours at room temperature and then treated with BODIPY 493/503 (Thermo Fisher Scientific, D3922) for 10 minutes. Finally, the tissues were washed with PBS and mounted onto glass slides for imaging. For retina staining, eyeballs were isolated and fixed in 3.7% PFA on ice for 10 minutes. Retinas were dissected and fixed in 3.7% PFA for another hour. Then the retina was washed in PBS, incubated in 5% BSA and 0.3% Triton X-100 for blocking and permeabilization for 1 hour at room temperature, and incubated with IsoB4 AF594 (Invitrogen I21413) and BODIPY 493/503 (Thermo Fisher Scientific, D3922) for 2 hours. Finally, the retina was washed with PBS and mounted onto glass slides for imaging.

### Body composition analysis.

Body weight and body composition were measured in male mice using the EchoMRI 3-in-1 system nuclear magnetic resonance spectrometer (Echo Medical Systems).

### Plasma lipid assays.

Collected plasma samples were subjected to TG assay (Fisher Scientific, TR22421), glycerol assay (MilliporeSigma, F6428), cholesterol assay (Invitrogen, A12216), and FFA assay (MilliporeSigma, mak044) according to the manufacturer’s instructions.

### Glucose-tolerance test.

Twenty-week-old male mice were fasted for 6 hours and weighed, and a baseline blood glucose level was measured using a glucometer. Each mouse was injected with 2 g/kg of glucose solution i.p. Blood glucose was measured at indicated time points.

### Nitrate and nitrite measurements.

Blood was collected from the ad libitum–fed male and female mice between 7 and 11 am. Plasma was collected by spinning whole blood at 3,000*g* for 15 minutes at 4°C and filtered through an Amicon 10K filter at 16,000*g* for 15 minutes at 4°C. Total nitrate and nitrite levels were measured by using the Nitrite/Nitrate Assay Kit (MilliporeSigma, 23479) following the manufacturer’s instructions.

### Pressure myograph to measure vasodilatory function.

Vasoreactivity was measured in male mice by using DMT 114P pressure myography. Mice were sacrificed by CO_2_ asphyxiation and the left carotid artery was immediately removed, stripped of fat, and kept in HBSS in a 37°C incubator until it was secured to cannulas using silk sutures. Mounted vessels in the pressure myograph chamber were filled with warm HBSS and were visualized by light microscopy. The vessels were then pressurized gradually to 40, 60, and 100 mmHg. Arteries were preconditioned by gradually increasing pressure to 100 mmHg with HBSS and then preconstricted by phenylephrine (10^–5^M). Then the vessels were subjected to cumulative concentrations of acetylcholine (ACh) (10^–9^M to 10^–4^M) for vasodilation. Vessel diameter change was continuously monitored using MYOVIEW II software (DMT).

### PCSK9 overexpression to induce atherosclerosis.

A liver-targeted gain-of-function PCSK9 model was used to induce atherosclerosis (48). AAV8-PCSK9 injection leads to LDL receptor (LDLR) KO-like phenotype, reducing hepatic uptake of LDL by increasing the lysosomal degradation of LDLRs. Groups of 8 mice from WT versus *Atgl* ECKO mice were tail vein injected with 5 × 10^11^ vector genomes of the virus and provided a Western diet for 12 weeks. Plasma cholesterol levels were measured at 3 and 10 weeks during the study period. At the end of 12 weeks, the aorta from the ascending aorta and aortic arch to thoracic aorta were dissected for Oil Red O staining to visualize lipid buildup within the aorta (49). Quantification of lipid buildup within the aorta was performed using ImageJ software (NIH) and normalized to total vessel area. Liver tissues were collected to confirm the reduction of LDLR protein by Western blot (WB).

### Cytokine array from cell culture media and mouse plasma.

Proteome Profiler Human Cytokine Array Kit (R&D Systems, ARY005B) was used for the media samples collected from HUVECs. Proteome Profiler Mouse Cytokine Array Kit (R&D Systems, ARY006) was used for the mouse plasma samples.

### EC culture.

Pooled HUVECs were purchased from Lonza and used between passages 3 and 6. Cells were grown in EBM2 containing EGM supplements (Lonza, CC-3162) with 10% FBS. Fully confluent HUVECs were subjected to siRNA transfection by using Lipofectamine RNAiMAX Reagent (Invitrogen) for knockdown studies. Cells were kept in serum-free Opti-MEM media for 5 to 6 hours of transfection duration, after which they were refreshed with 10% EGM2. All siRNAs were treated at 10 nM concentration and were obtained from Sigma-Aldrich: human siATGL (SASI_225605), mouse siATGL (SASI_33377), human siDGAT1 (SASI_77408), human siACSL1 (SASI_202187), human siCPT1 (SASI_231321), human siRELA (SASI_171091), human siRELB (SASI_103187), human siP50 (SASI_181061), human siEEF1A1 (SASI_331771), human siPTBP1 (SASI_216643, 216644), and human siPCBP1 (SASI_34329). Confirmation of siRNA-mediated genetic knockdown was determined by using multiple different methods, including qPCR, WB, or immunocytochemistry (ICC). Drugs that were used were obtained from either MilliporeSigma or MCE: forskolin (Sigma, F6886), iDgat A922500 (MilliporeSigma, A1737), atglistatin (MilliporeSigma, SML 1075), MG132, WY14643 (MilliporeSigma, pirinixic acid, PPARɑ/γ agonist, C7081), rosiglitazone (MilliporeSigma, PPARγ agonist, R2408), fenofibrate (MilliporeSigma, PPARɑ agonist, F6020), pemafibrate (MCE, HY-17618), raspberry ketone (MCE, HY-N1426), clofibrate (MilliporeSigma, 6643), and bezafibrate (MilliporeSigma, 7273).

### Mouse primary EC isolation.

Aorta from 3 mice were dissected and digested in 2 mg/mL type I collagenase and dispase in serum-free DMEM for 30 minutes; 10% FBS DMEM was added to quench collagenase/dispase, and the tissue homogenates were then titrated and filtered through 100 μm and 40 μm filters. After washing with PBS and centrifugation at 300*g* for 5 minutes, cell pellets were resuspended in 0.5% BSA in PBS and incubated with magnetic bead conjugated with anti-CD31 antibody (BD Biosciences — Pharmingen, 553370) for 15 minutes with gentle mixing on ice. CD31-bound ECs were collected after vigorous washing using a magnetic bar.

### qPCR.

mRNA isolation and cDNA synthesis were done by using the TurboCapture mRNA Kit (QIAGEN) according to the manufacturer’s instructions. qPCR was performed on the CFX384 Bio-Rad Real-Time PCR Detection System using SYBR Green. Sequences of the primers used in this study were as follows: human ATGL (forward: ATGGTGGCATTTCAGACAACC, reverse: CGGACAGATGTCACTCTCGC), mouse ATGL (forward: CAACGCCACTCACATCTACGG, reverse: GGACACCTCAATAATGTTGGCAC), human eNOS (forward: GAGACTTCCGAATCTGGAACAG, reverse: GCTCGGTGATCTCCACGTT), mouse eNOS (forward: TCAGCCATCACAGTGTTCCC, reverse: ATAGCCCGCATAGCGTATCAG), human Dgat1 (forward: TATTGCGGCCAATGTCTTTGC, reverse: CACTGGAGTGATAGACTCAACCA), human ACSL1 (forward: CGACGAGCCCTTGGTGTATTT, reverse: GGTTTCCGAGAGCCTAAACAA), human CPT1 (forward: TCCAGTTGGCTTATCGTGGTG, reverse: TCCAGAGTCCGATTGATTTTTGC), and human MCP1 (forward: CAGAAGTGGGTTCAGGATTCC, reverse: ATTCTTGGGTTGTGGAGTGAG).

### WB.

Cells were lysed in RIPA buffer that contained phosphatase inhibitor (PhosSTOP, Roche) and proteinase inhibitor (cOmplete Mini Proteinase Inhibitor Cocktail, Roche). The insoluble cell debris or lipid fraction was removed by centrifugation at 16,000*g*. Protein concentration was measured with the BCA Protein Assay Kit (Thermo Fisher Scientific). Samples were then boiled in SDS sample buffer and loaded into 4% to 20% gradient gel (Bio-Rad), transferred to PVDF membrane (Millipore), and analyzed by immunoblotting. The following antibodies were used: ATGL (CST2138), eNOS (CST32027), 14-3-3 (CST8312), p-eNOS S1177 (BD 612392), LDLR (10785-1-AP), PLIN2 (abcam 108323), GAPDH (CST5174), HDAC2 (CST5113), KDEL (abcam12223), COXIV (CST11967), Tomm20 (BD 612278), and TFAM (abcam 119684). Secondary antibodies were purchased from Cell Signaling Technology. Signal was detected using the ECL System (ImageQuant LAS 4000, Amersham Biosciences, GE Healthcare) according to the manufacturer’s instructions.

### ICC.

Confluent HUVECs were plated onto glass coverslips and subjected to siRNA transfection and/or drug treatment. Samples were fixed with 3.7% paraformaldehyde, washed, and permeabilized with 0.3% Triton X-100. For PCBP1 staining, cells were permeabilized with 0.05% digitonin. After blocking with 2% BSA, samples were incubated with primary antibodies overnight and with secondary antibodies for 2 hours at room temperature, then treated with BODIPY 493/503 at 0.1 mg/mL for 10 minutes. Finally, they were washed and mounted onto glass slides using VECTASHIELD for imaging. For flow cytometry analysis of BODIPY staining, HUVECs that were knocked down and/or treated with drugs were incubated with BODIPY while adherent; 0.25% trypsin was used to lift cells off of dishes, and 0.1% BSA was used to wash them before centrifugation. Cells were resuspended in 0.1% BSA and kept on ice until flow cytometry analysis using Accuri (BD Biosciences).

### RNA-Seq and GO analysis.

Total RNAs from HUVECs that were knocked down with siCTL versus siATGL were isolated using TRIzol and the QIAGEN RNeasy kit. Ribosomal RNAs were depleted by polyA selection prior to library prep. The samples were then sequenced on an Illumina HiSeq in a 2 × 150 bp paired end configuration with 20 to 30 million reads per sample. Samples were randomized and handled in a blinded fashion during sample preparation and sequencing. RNA-Seq reads were aligned to the human reference genome using STAR (version 2.7.9a) (50). Picard (https://broadinstitute.github.io/picard/) was used to mark the duplicates, collect the RNA-Seq metrics, and estimate the library complexity. The raw read counts were computed using the Rsubread package (version 2.0.3) (51) and normalized into counts per million (CPM) using the CPM function from the edgeR package (version 3.1.4) (52). The genes with 25% of samples with a CPM of less than 1 were deemed as low expressed and removed from further analysis. Data were also transformed using the VOOM function from the Limma package (53). Differential gene expression analysis was performed using the lmFit function from the Limma package. Top 3,000 differentially expressed genes were used for Molecular Function GO analysis by using ShinyGO, version 0.741 (30).

### LD isolation.

Endothelial LDs were isolated based on published methods with modifications (20, 54). Ten 15 cm dishes of confluent HUVECs were pooled for each LD purification. Harvested cells were washed twice with ice-cold PBS and lysed in hypotonic lysis buffer (HLM) (20 mM Tris-Cl, pH 7.4, 1 mM EDTA, protease inhibitor and phosphatase inhibitor) by pipetting the cells up and down. The suspended cells were incubated on ice for 10 minutes. Cell lysates were then further dounced 40 times with tissue grinder using a loose pestle (Wheaton). Cell debris and unlysed cells were removed by centrifugation at 600*g* at 4°C for 5 minutes. The remaining lysates were centrifuged at 3,000*g* at 4°C for 30 minutes to remove nuclei. Postnuclear fraction was centrifuged at 20,000*g* to remove other intracellular organelles including Golgi, ER, mitochondria, and peroxisomes. The supernatant was collected and adjusted to 20% sucrose in HLM by adding one-third volume of 60% sucrose HLM. Lysate in 20% sucrose HLM was layered into the bottom of an ultracentrifuge tube for an SW41Ti rotor. The same volume of 5% sucrose HLM was layered over the sample. More HLM of the same volume was layered over the sucrose layers. The sucrose gradient tubes were centrifuged at 28,000*g* at 4°C for 30 minutes. LD fraction was collected from the top of the tube and was delipidated by incubating in acetone on dry ice, followed by centrifugation for 1 hour at 4,000*g* at 4°C. The pellet was dried under a gentle stream of nitrogen and resuspended in protein lysis buffer. The isolated LD protein underwent WB followed by MS analysis. For proteomics analysis, the samples were separated using SDS-PAGE, and the entire gel section was excised and submitted to the Harvard Taplin Mass Spectrometry Facility (Boston, Massachusetts, USA) for in-gel digestion of gel bands, microcapillary liquid chromatography with tandem MS (LC/MS/MS) analysis, protein database searching, and data analysis.

### LD proteomics data set analysis.

To compare the LD proteome across 3 different cell types (U2OS, Huh7, and HUVECs), we utilized the gProfiler MultiQuery Analysis feature. The analysis was performed using a specific workflow and data set, which can be accessed at g:Profiler (http://biit.cs.ut.ee/gplink/l/2dmu9ASPQt). The output of this analysis provides information on the similarities and differences in the functional properties of LD-associated proteins among the 3 cell types (full data set in Supplemental Table 2). A web-based tool, DeepVenn (https://www.deepvenn.com/), was used to create Venn diagrams of 3 data sets on the “metabolism of lipids” pathway (Figure 5C). Enrichr was used for GO analysis of biological process in the LD proteome data set from HUVECs (full data set in Supplemental Table 3). The top 5 of the GOs ranked by adjusted *P* value are highlighted.

### Mendelian randomization and colocalization.

Two-sample Mendelian randomization using summary statistics was performed using the TwoSampleMR (55) package in R. Genetic instruments were constructed using conditionally independent expression quantitative trait loci (eQTLs) for genes encoding proteins involved in LD formation/degradation (*PNPLA2*, *GPAM*, *AGPAT1*, *AGPAT3*, *AGPAT5*, *LPIN1*, *LPIN2*, *DGAT1*, *DGAT2*, *LIPE*, and *MGLL*) in vascular tissues (aorta, coronary artery, and tibial artery) from GTEx, version 8 (35, 36). Corresponding effects for each variant on SBP and DBP were obtained from the International Consortium of Blood Pressure Genome Wide Association Studies (ICBP) and UKB GWAS metaanalysis, which included up to 757,601 participants of European ancestry. After harmonization of effect alleles, Mendelian randomization was performed using the inverse variance weighted method with multiplicative random effects (when multiple genetic variants associated with gene expression were present) or the Wald ratio method (when a single genetic variant associated with gene expression was present). *P* values of less than 0.05 for 24 gene-tissue pairs were considered significant. Bayesian enumeration colocalization was performed as a sensitivity analysis for significant associations using the coloc package in R (37, 56). Genetic variants ± 250 kb surrounding the *PNPLA2* locus were obtained from GTEx, version 8, with summary statistics deposited in the eQTL catalog and the ICBP+UKB BP GWAS (35, 36, 57). Given the sensitivity of enumeration colocalization to the specified Bayesian priors, we considered a range of priors reflecting varying anticipated probabilities of a shared causal variant influencing both gene expression and BP. We considered both the default prior (*P*_12_ = 1 × 10^–5^) and a range of more skeptical and more optimistic priors (justified by the experimental findings in mice).

### Statistics.

*P* values were calculated using 2-tailed Student’s *t* test. For statistical comparisons between study groups, 1-way ANOVA was used, followed by Bonferroni’s post hoc testing. *P* < 0.05 was considered statistically significant. All data are represented as mean ± SD. Data from cell culture experiments are representative from a minimum of 3 independent experiments.

### Study approval.

All mouse experiments were performed according to procedures approved by the University of Pennsylvania Institute for Animal Care and Use Committee.

### Data availability.

RNA-Seq data have been deposited in the NCBI’s Gene Expression Omnibus database (GEO GSE231619). The full data set from LD proteomics analysis is provided in Supplemental Table 1. Values for all data points in graphs are reported in the Supporting Data Values file.

## Author contributions

BK led the studies and was directly involved in most experiments. ZA oversaw the studies. BK, WZ, SYT, MGL, AI, YY, ER, LL, and JL conducted experiments and acquired data. BK, RKA, GAF, and ZA interpreted data. BK and ZA wrote the manuscript. All authors discussed the results and commented on the manuscript.

## Supplementary Material

Supplemental data

Supplemental table 1

Supplemental table 2

Supplemental table 3

Supporting data values

## Version 1 10/12/2023

In-Press Preview

## Version 2 12/15/2023

Electronic publication
