# Supplementary material for: Endothelial lipid droplets suppress eNOS to link high fat consumption to blood pressure elevation
Source: J Clin Invest. 2023 Dec 15;133(24):e173160. doi: 10.1172/JCI173160 (PMC10721151; doi:10.1172/JCI173160)
Supplement: Supplemental data [file jci-133-173160-s172.pdf]

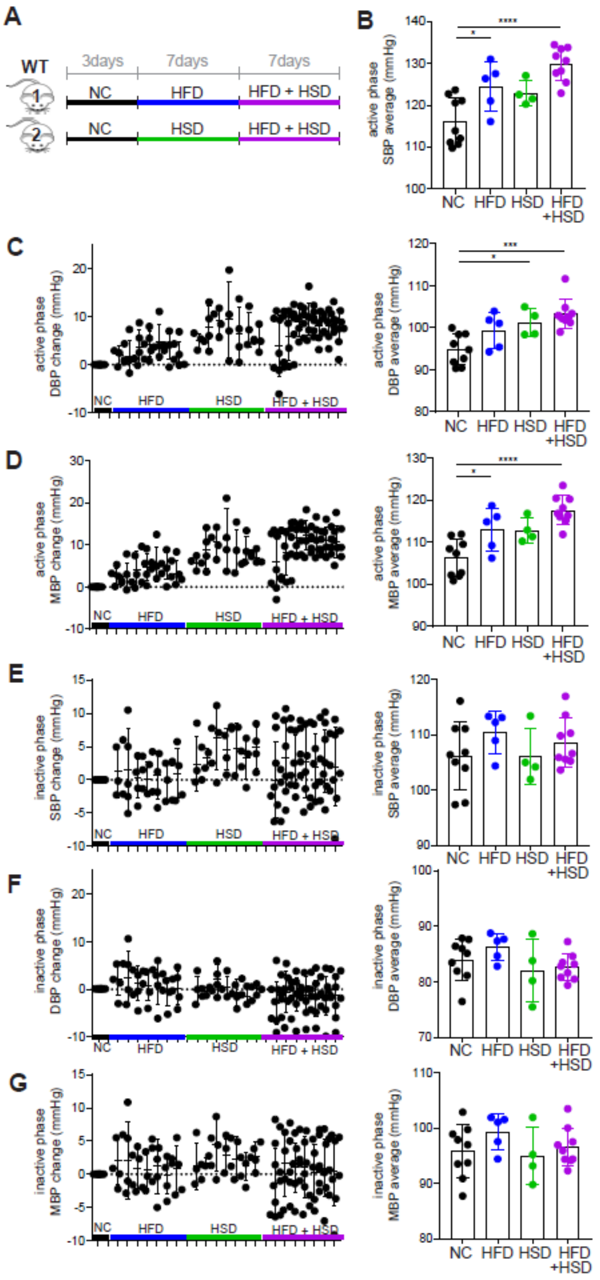

Supplementary Figure1. High fat diet induces blood pressure elevation during active phase.

A. Experimental setup for administration of normal chow (NC), high fat diet (HFD), high salt diet (HSD), or both HFD+HSD in wild type C57BL/6J mice, while monitoring blood pressure by non-invasive telemetry. **B**. Average active phase SBP while provided with the indicated diet. **** $p < 0.0001$, * < 0.05 by 1-way ANOVA. **C**. Left panel: Elevation of diastolic blood pressure (DBP) during active phase. Right panel: Average active phase DBP. *** $p < 0.001$, * < 0.05 by 1-way ANOVA. **D**. Left panel: Elevation of mean blood pressure (MBP) during active phase. Right panel: Average active phase MBP. **** $p < 0.0001$, * < 0.05 by 1-way ANOVA. **E**. Left panel: Elevation of SBP during inactive phase. Right panel: Average inactive phase SBP. **F**. Left panel: Elevation of DBP during inactive phase. Right panel: Average inactive phase DBP. **G**. Left panel: Elevation of MBP during inactive phase. Right panel: Average inactive phase MBP.

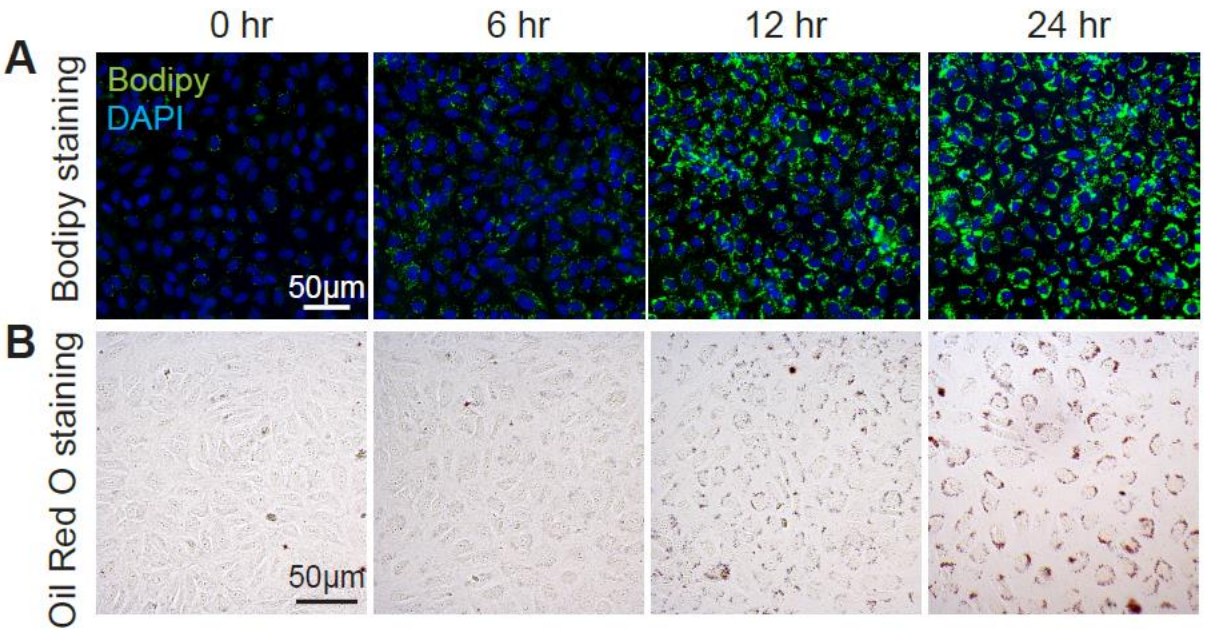

Supplementary Figure2. Fatty acids induce endothelial lipid droplet accumulation in vitro. **A-B**. Time course incubation of 500 μM oleic acid (OA) in HUVECs. Lipid droplets were visualized with bodipy (A) or Oil Red O (B) staining.

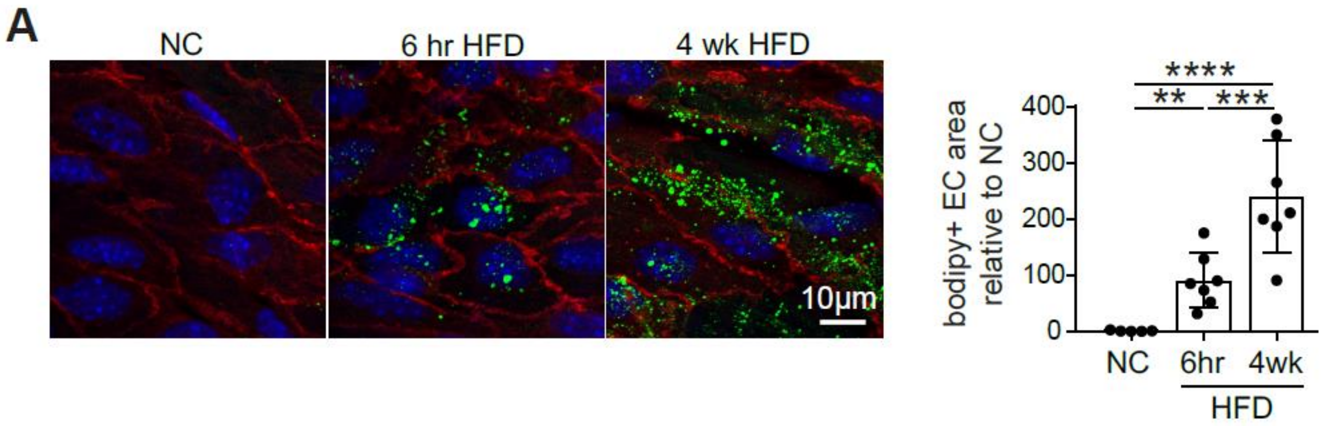

Supplementary Figure3. Long-term high fat diet (HFD) induces greater lipid droplet accumulation in the endothelium in vivo. Left panel: *en face* staining of thoracic aorta upon normal chow (NC), 6-hour HFD, or 4-week HFD using bodipy (green), CD31 (red), and DAPI (blue). Right panel: bodipy-stained area in the endothelium is quantified. **** $p < 0.0001$, *** < 0.001, ** < 0.01 by 1-way ANOVA.

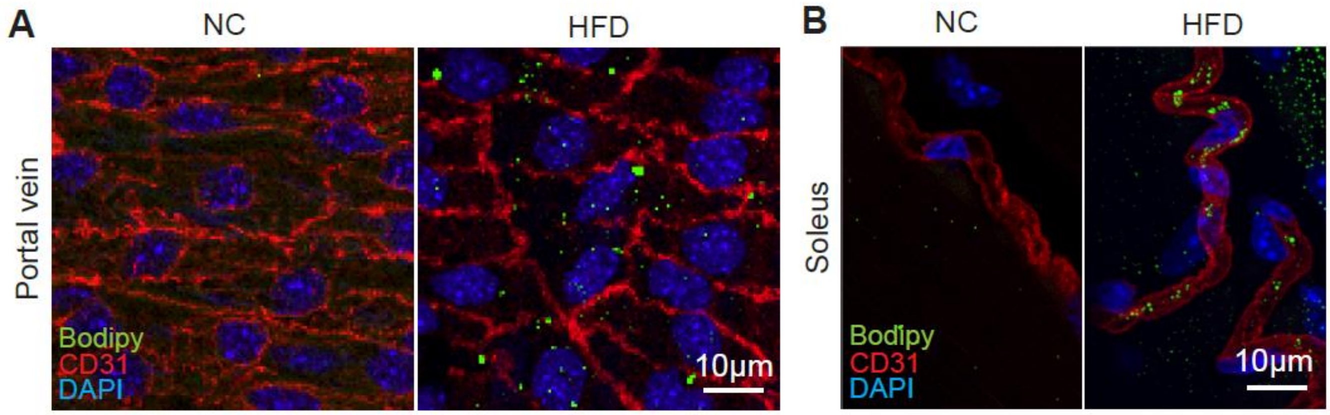

Supplementary Figure4. High fat diet induces endothelial lipid droplet accumulation in vivo. A. *en face* staining of portal vein using bodipy (green), CD31 (red), and DAPI (blue), after 5 hours of either NC or HFD *ad lib* feeding. **B.** Whole-mount staining of capillary vessel in the soleus using bodipy (green), CD31 (red), and DAPI (blue), after 5 hours of either NC or HFD *ad lib* feeding.

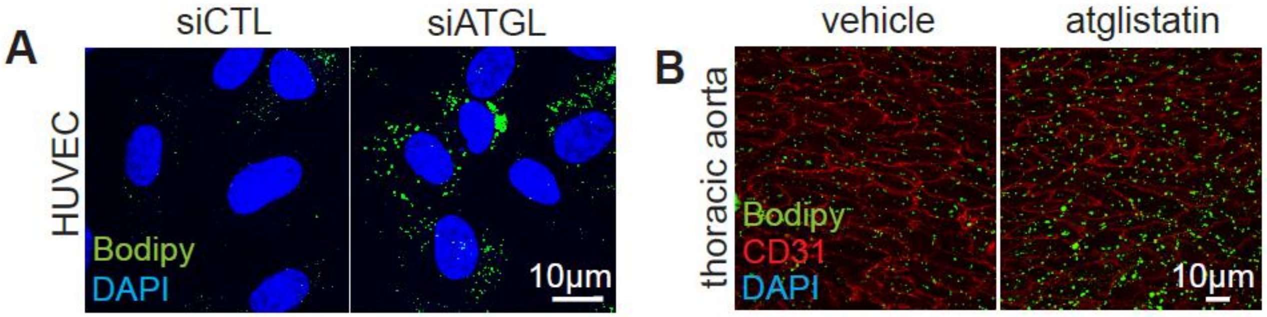

Supplementary Figure5. Suppression of ATGL leads to the accumulation of lipid droplet in endothelial cells. **A**. Immuno-staining of HUVECs treated with control or *Atgl* siRNA for 2 days. Bodipy (green) and DAPI (blue). **B**. *en face* staining of the thoracic aorta after 24 hours of *ex vivo* culture in the presence of vehicle or atglistatin (ATGL inhibitor). Bodipy (green), CD31 (red), and DAPI (blue).

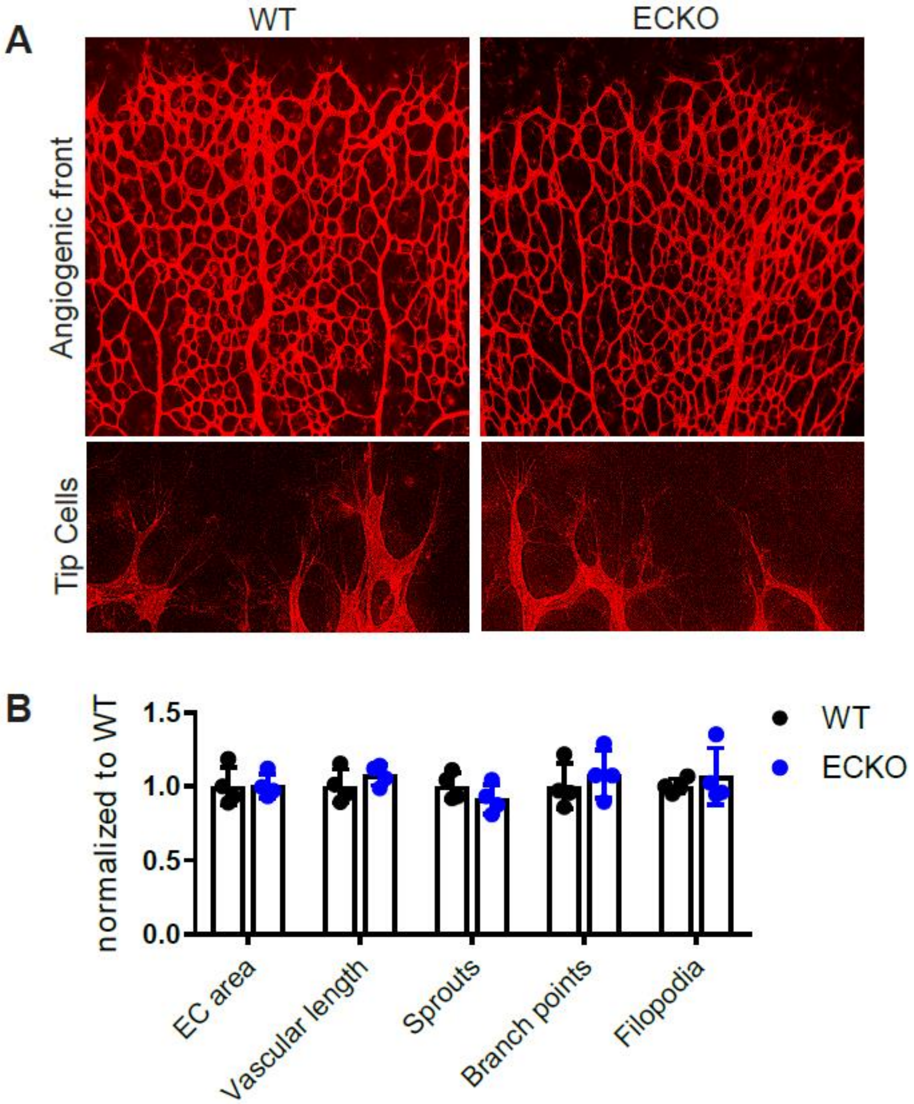

Supplementary Figure6. *Atgl* ECKO mice exhibit normal retinal vascular development. A. Whole-mount staining of retina vasculature at postnatal day 5 with IsoB4 (red). Representative images from angiogenic front (upper panel) and tip cells (lower panel) are shown. **B**. Quantification of the endothelial cell area, vascular length, number of sprouts, number of branch points, and number of filopodia are indicated.

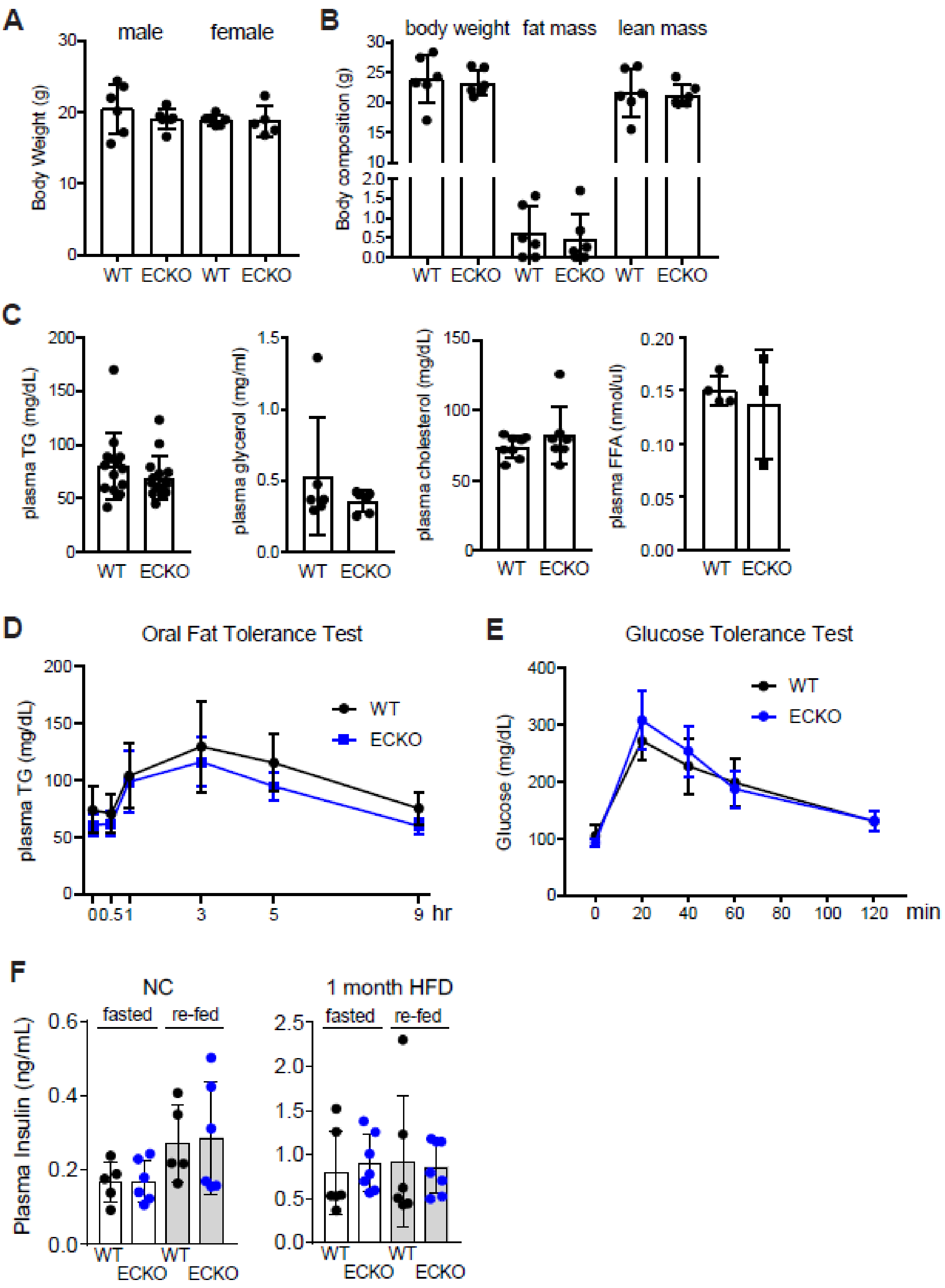

Supplementary Figure7. *Atgl* ECKO mice appear grossly and metabolically normal. A. Body weight of the mice at 12-wk-old age. **B.** Body composition measured by EchoMRI at 20-wk-old of male mice. **C.** Plasma lipid profiles including triglyceride (TG), glycerol, cholesterol, and FFA after 5 hours of fasting. **D.** Oral fat tolerance test in 20-wk-old male mice. Plasma TG was measured from tail blood at the indicated time points after administration by oral gavage of olive oil (10 ml/kg). **E.** Glucose tolerance test in 20-wk-old male mice. Glucose from tail blood was measured at the indicated time points after IP injection of glucose (2 g/kg). **F**. Plasma insulin levels measured in 20-week-old male mice under fasted and refed conditions after being maintained on either a normal chow (NC) or high-fat diet (HFD) for one month.

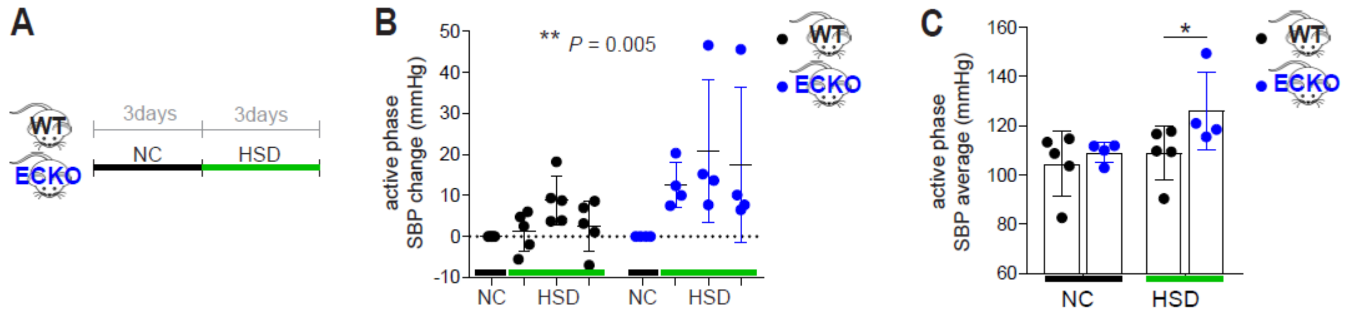

Supplementary Figure8. *Atgl* ECKO mice are susceptible to salt-induced blood pressure elevation. A. Experimental setup for the administration of NC or high salt diet (HSD) in WT vs *Atgl* ECKO mice. **B**. Elevation of SBP during active phase while provided with the indicated diet for 3 days. N=5 each genotype. ** $p < 0.01$ by 2-way ANOVA. **C**. Average active phase SBP. * $p < 0.05$ by 1-way ANOVA.

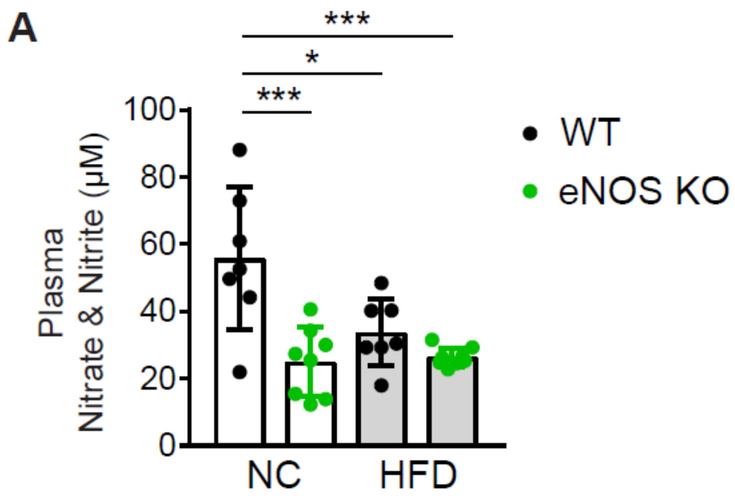

Supplementary Figure9. eNOS knockout (KO) animals do not exhibit changes in their plasma NO levels in response to high fat diet (HFD). **A**. Nitrate and nitrite levels measured in plasma from WT and eNOS KO mice receiving either normal chow (NC) or 6 weeks of HFD. *** $p < 0.001$, * < 0.05 by 1-way ANOVA. N = 7-8 mice/group.

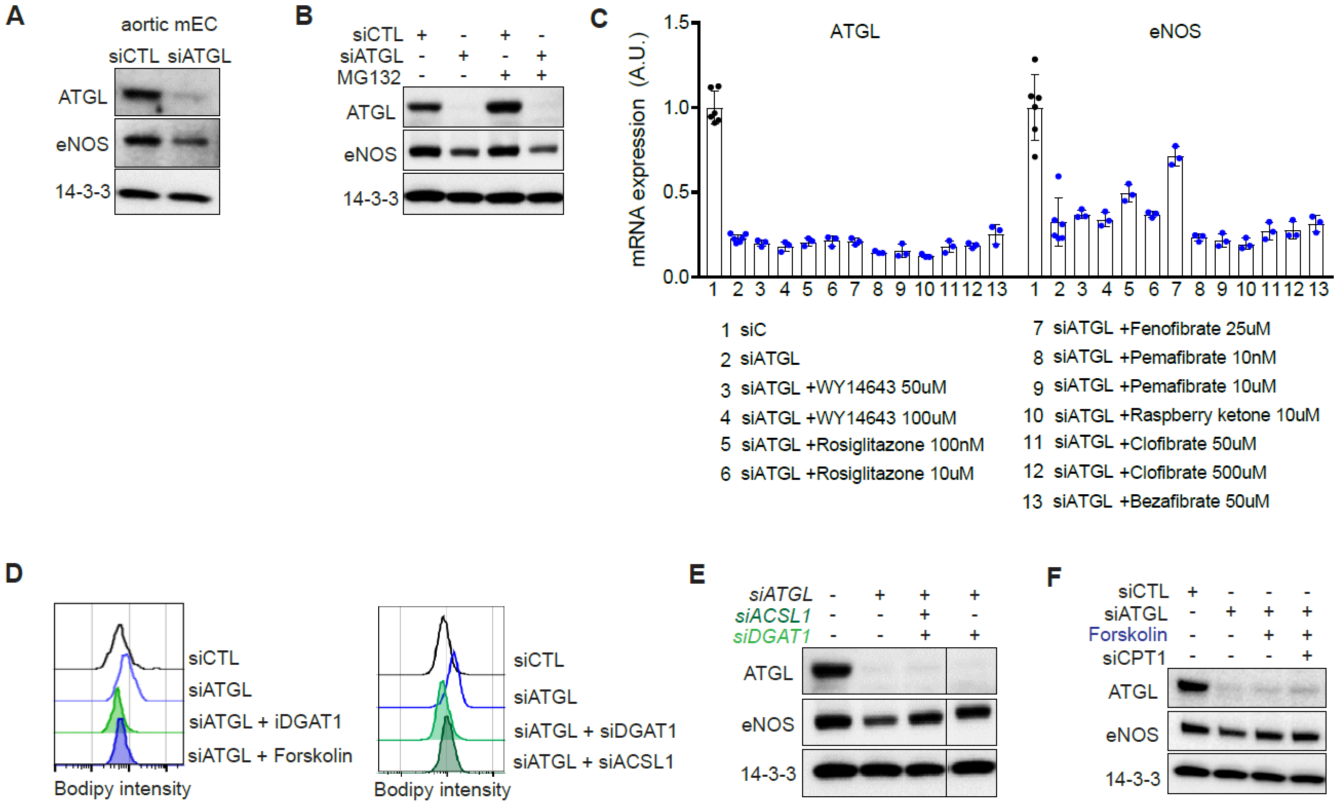

Supplementary Figure10. ATGL knockdown suppresses eNOS and normalization of LD contents restores eNOS. A. WB of ATGL and eNOS in primary cultured aortic mouse ECs treated with siATGL vs siCTL for 2 days. **B**. WB of ATGL and eNOS in HUVECs treated with siATGL vs siCTL for 2 days and with MG132 during the last 6 hour, as indicated. **C**. qPCR analysis of ATGL and eNOS in HUVECs treated with siATGL and with the indicated PPAR agonists at the indicated concentrations for 2 days. **D**. Flow cytometry analysis of bodipy intensity in HUVECs 2 days after indicated siRNA or drug treatment. **E**. WB of ATGL and eNOS in HUVECs treated with combinations of siACSL1, siDGAT1, or siATGL for 2 days, as indicated. **F**. WB of ATGL and eNOS in HUVECs treated with combinations of siCPT1, siATGL, or forskolin, as indicated.

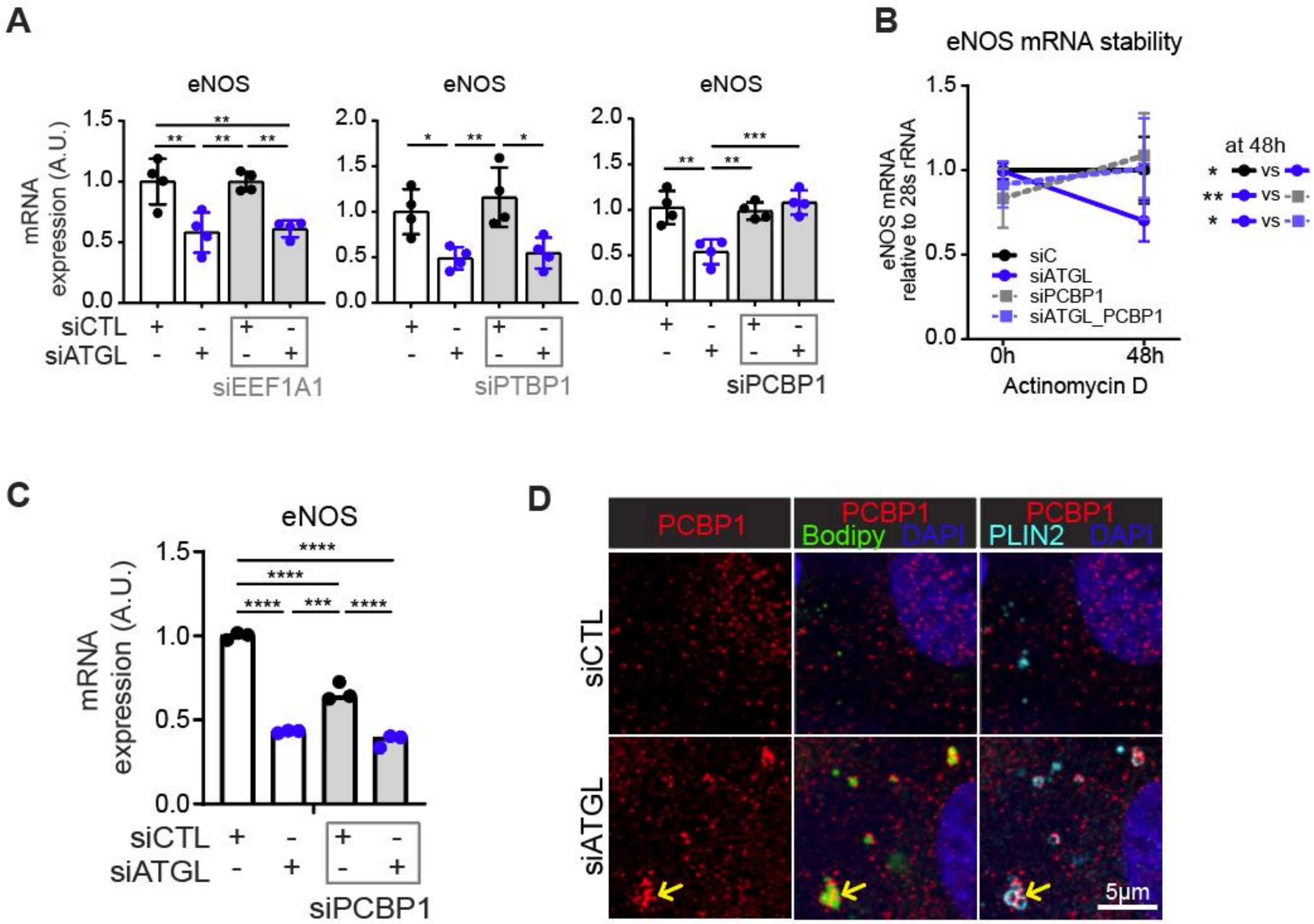

Supplementary Figure11. PCBP1 as a potential regulator of eNOS mRNA stability. A. Quantification of eNOS mRNA by qPCR in HUVECs treated with the indicated siRNAs for 48 hours. HUVECs were co-transfected for 48 hours with si-EEF1A1, -PTBP1, or -PCBP1 and either siCTL or siATGL. *** $p < 0.001$, ** < 0.01, * $p < 0.05$ by 1-way ANOVA. **B**. eNOS mRNA stability measurements in HUVECs with indicated siRNA knockdown in response to Actinomycin D treatment (5nM). eNOS mRNA levels at indicated time points following Actinomycin D were normalized to 28s rRNA. ** < 0.01, * $p < 0.05$ by 2-way ANOVA. **C.** Quantification of eNOS mRNA by qPCR in HUVECs treated with the indicated siRNAs for 96 hours. HUVECs were co-transfected with si-PCBP1 and either siCTL or siATGL. **** $p < 0.0001$, *** $p < 0.001$ by 1-way ANOVA. **D**. Immuno-staining of HUVECs treated with control or Atgl siRNA for 2 days. PCBP1 (red), PLIN2 (cyan), Bodipy (green) and DAPI (blue).

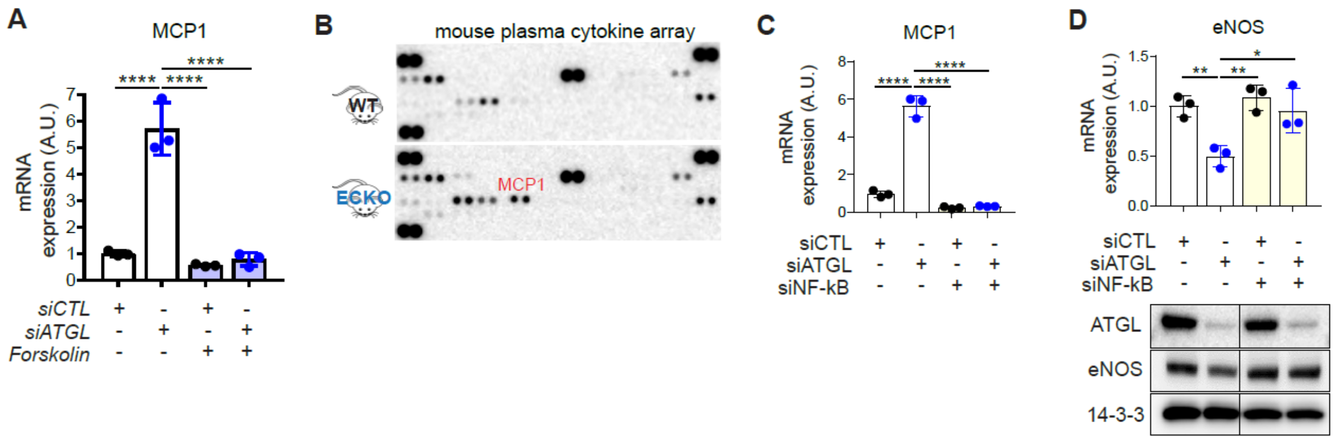

Supplementary Figure12. Suppression of ATGL induces MCP1 in a NF-kB-dependent manner. A. Quantification of MCP1 mRNA by qPCR following siATGL vs siCTL for two days with and without Forskolin treatment. **B**. Cytokine array assay with plasma collected from WT vs *Atgl* ECKO mice. **C**. Quantification of MCP1 mRNA by qPCR following siATGL vs siCTL for two days with or without siNF-kB. **D**. Quantification of eNOS by qPCR (upper panel) and WB (lower panel) following siATGL vs siCTL for two days with or without siNF-kB. **** $p < 0.0001$, ** < 0.01, * < 0.05 by 1-way ANOVA.

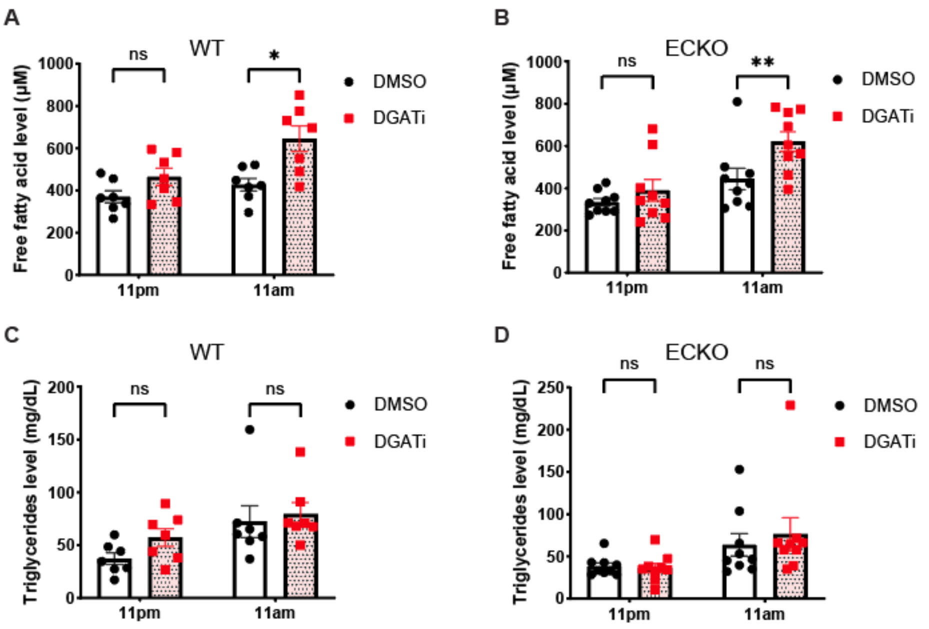

Supplementary Figure13. Dgat1 inhibitor does not suppress post-prandial lipemia. A-B. Quantification of plasma free fatty acids in WT (A) and *Atgl* ECKO (B) mice at the indicated time point after 3-days of DMSO vs iDgat1 injection. iDgat1 was given at 3mg/kg via IP injection. B-C. Quantification of plasma triglycerides in WT (C) and *Atgl* ECKO (D) mice at the indicated time point after 3-days of DMSO vs iDgat1 injection. iDgat1 was given at 3mg/kg via IP injection. ** < 0.01, * < 0.05 by 1-way ANOVA.
